# A systematic review of elephant impact across Africa

**DOI:** 10.1371/journal.pone.0178935

**Published:** 2017-06-07

**Authors:** Robert A. R. Guldemond, Andrew Purdon, Rudi J. van Aarde

**Affiliations:** Conservation Ecology Research Unit, Department of Zoology and Entomology, University of Pretoria, Pretoria, South Africa; University of Tasmania, AUSTRALIA

## Abstract

Contradictory findings among scientific studies that address a particular issue may impede the conversion of science to management implementation. A systematic review of peer-reviewed studies to generate a single outcome may overcome this problem. The contentious topic of the impact that a megaherbivore such as the savanna elephant have for other species and their environment can benefit from such an approach. After some 68 years, 367 peer-reviewed papers covered the topic and 51 of these papers provided sufficient data to be included in a meta-analysis. We separated the direct impact that elephants had on trees and herbs from the indirect effects on other vertebrates, invertebrates, and soil properties. Elephants have an impact on tree structure and abundance but no overall negative cascading effects for species that share space with them. Primary productivity explained a small amount of variation of elephant impact on vegetation. Elephant numbers (density), study duration, rainfall, tree cover, and the presence of artificial water and fences failed to describe patterns of impact. We conclude that published information do not support the calls made for artificially manipulating elephant numbers to ameliorate elephant impact, and call for the management of space use by elephants to maintain savanna heterogeneity.

## Introduction

The impact that iconic megaherbivores have on their environment and on other species carries considerable emotion and disagreement [1–2]. Park managers with a mandate to maintain biodiversity often face disagreements in the scientific literature on how to deal with the consequences of megaherbivore impact [3]. Consequently, to formulate policy they sometimes have to rely on experience and personal opinions [4] instead of scientific evidence [5]. Often, an option is to consider population control to curb impact, which creates great societal discord [2]. A systematic review of the scientific literature may empower conservation managers with evidence-based decisions, by combining the results of relevant and independent peer-reviewed studies to strengthen overall trends on impact and clarify some of the possible discrepancies among peer-reviewed studies [6].

Megaherbivores the world over have disproportional effects on their environment [7–8]. Such is the case with the savanna elephant (*Loxodonta africana* Blumenbach 1797) that dominates mammalian biomass in sub-Saharan savannas [9] and that can transform landscapes to the detriment of other species and biological diversity [10–12]. Elephants, however, may also contribute to the maintenance of these savannas and benefit some species that share space with them [13–15]. Elephants, like other large herbivores such as deer [16], European bison [17], and the sika deer in Japan [18] can change the plant structure especially where they occur at high densities [19]. Nevertheless, not all of the many published studies agree on the impact of elephants on vegetation and on the possible cascading and negative consequences for other species [3,20]. Most studies, however, agree that elephants in African can change the savanna landscape [21] and that confinement through fencing and by providing water impairs roaming that may accentuate impact [22], which sometimes are to the detriment of other species and vegetation structure [11]. We therefore opted to extend our previous meta-analysis on elephant impact on savanna vegetation [3], to seek for evidence of cascading effects on other species [7,23], and to describe co-variates of impact as a step towards addressing the management of elephants.

Most conservation managers conceded that elephants change the structure of vegetation, especially where they occur in relatively high densities in protected areas, and when elephants (and other game species) receive water artificially under fenced conditions [11]. These changes to vegetation are then assumed to be detrimental to the maintenance of biodiversity, which is the mandate set for conservation officials. An explanatory variable from which to evaluate impact often include elephant numbers [10], and ignore the prevailing conditions under which elephants find themselves such as the dynamic nature of savannas (rainfall, primary productivity, tree cover), and management legacies by providing water and fencing in some of the protected areas [3].

We address two pertinent issues in this paper. What does the scientific literature tell us about the impact that elephants have for savanna vegetation and co-occurring species? What drives the apparent impact of elephants? We followed the protocols described for a systematic review to source, evaluate, and extract the data from peer-reviewed studies done across the African continent. We expected elephants to have a negative impact on trees and shrubs and that this impact is a function of elephant numbers, time (years), rainfall, primary productivity, the provision of water, and the presence of fences.

## Methods

We searched for scholarly papers on elephant impact using the *Biological Sciences*, *Scopus*, *Zoological Record* and *Wildlife Ecology and Studies Worldwide* search engines. Our search terms were “elephant*”, “tree*”, “vegetation”, “biodiversity*”, “damage”, ‘impact” in the titles, abstracts and keywords of English written papers published up to December 2015 (final search was in April 2016).

We excluded books, book chapters, conference proceedings, technical reports, preprints [24] and unpublished data. Postgraduate theses sometimes have findings published in the formal literature and were therefore not considered to avoid possible duplication. We used the maps from [25–26] to select only the published studies done at study sites with savanna elephants [*L*. *africana*] and excluded those studies from sites containing forest elephants [*L*. *cyclotis* Matschie 1900]. We excluded papers that reported on elephant in wildlife sanctuaries, or where reported impact was on exotic vegetation. In total we found 367 papers on elephant impact, of which we had to disregard 294 papers from further consideration because these lacked data (e.g. opinion papers, narrative reviews), consisted of secondary analyses of primary data, or failed to report the minimum statistical information for meta-analysis (e.g. *n* or *df*, mean, SD or SE). Of the remaining 73 papers, 51 of them compared variables in the presence and absence of elephants (e.g. elephant exclosures, or inside vs outside protected areas). The remaining 22 papers reported on elephant impact in observational studies with no controls in place, and we therefore did not consider these any further (see S1 Appendix).

For each selected paper, we recorded the author names and year of publication (study identity), journal details, study area and country, study duration (the number of years that elephants were excluded), study size (number of samples), details of the measurements (e.g. taxa, response variable) as well as the mean and variability values (SD or SE). We sourced the information from the text, tables, supplementary materials, or extracted the values from graphs using purpose designed-software (xyExtract V5.1).

Each paper used different variables, or combinations thereof to illustrate elephant impact. We separated the variables into direct and indirect effect classes, where the former include the response of trees, shrubs and herbs to browsing, grazing, knocking down trees, pollarding, breaking branches, stripping bark and feeding on roots [11]. The measurements here included *structural* changes to trees, shrubs, herbs (e.g. canopy size, stem diameter, tree height, grass cover and biomass), and changes in *abundance* (number of individuals, densities). The indirect effects for trees included *population* level responses (e.g. recruitment, survival, and mortality of adult tree or seedlings), changes in *diversity* indices (α- and β-diversity) and ecological *processes* linked to elephant browsing. Examples of such processes are the damage to trees inflicted by insects due to elephant browsing on those particular trees, thorn and spine growth responses, seed production, and changes to leaf polyphenols, tannins and protein levels ascribed to elephant feeding.

The responses of vertebrates (small mammals, birds and herpetofauna), invertebrates (ants, dung beetles, flies, butterflies and spiders), and soil properties (soil minerals, pH, silt, compaction, soil water content and infiltration) to the presence of elephants were analyzed separately as indirect impacts. The measurements relating to vertebrate and invertebrate responses were also grouped into *structural* (e.g. body weight of small mammals), *abundance* (e.g. number of individuals, density), *population* level responses (e.g. recruitment, survival), *diversity* indices (α- and β-diversity), and ecological *processes* (e.g. dispersal distances of small mammals, tree occupation by insects).

For each selected study, we sourced the elephant density at the time of the study (or the nearest time thereof), calculated a 15-year mean primary productivity using data layers of Enhanced Vegetation Index (EVI), extracted mean tree cover and mean annual precipitation (MAP). We also documented management interventions e.g. providing water artificially and erecting fences that blocked elephant movement (for details refer to S1 Table). We present a summary of the study site details and number of extracted values separately for each category for each site in Table 1.

**Table 1:** The 14 study sites ranked according the numbers of papers produced for each site, and the number of effects included in the meta-analysis.

Study site and country	Number of papers (*n*)	Elephant density (ind.km^-2^)	Primary productivity (0–1)	Tree cover (%)	Mean Annual Precipitation (mm.yr^-1^)	Management interventions	Number of effects (*k*) per study site	Number of effects (*k*) per category per study site					
								Structure	Abundance	Population	Diversity	Processes	Soil properties
1. Mpala Research Centre, Kenya	14	0·24 to 0·48	0·20	10·6	692	Water	214	36	113	42	11	12	—
2. Kruger National Park, South Africa	11	0·41 to 0·90	0·26	6·62	560	Both	233	22	106	1	47	3	54
3. Tembe Elephant Park, South Africa	6	0·38 to 0·60	0·37	15·8	750	Both	42	—	18	—	24	—	—
4. Addo Elephant National Park, South Africa	5	1·45 to 2·53	0·27	25.4	408	Both	34	2	19	—	2	5	6
5. Eastern Cape Private Reserves, South Africa	2	0·17	0·28	14·8	516	Both	11	5	6	—	—	—	—
6. Mana Pools, Zimbabwe	2	1·95	0·31	10·6	709	None	25	6	11	—	8	—	—
7. Phinda Private Game Reserve, South Africa	2	0·54	0·28	7·06	753	Both	6	—	4	—	2	—	—
8. Sweetwaters Game Reserve, Kenya	2	1·26	0·28	12·4	474	None	26	26	—	—	—	—	—
9. Amboseli Game Reserve, Kenya	1	0·27	0·17	1·04	685	None	13	13	—	—	—	—	—
10. Arabuko-Sokuke Forest Reserve, Kenya	1	0·44	0·45	41·2	693	Both	5	—	4	—	—	—	1
11. Endarakwai Ranch, Tanzania	1	-	0·19	3·56	692	Water	5	—	2	—	3	—	—
12. Kilombero Valley, Tanzania	1	1·03	0.35	16·2	1365	None	1	—	—	—	1	—	—
13. Murchison Falls National Park, Uganda	1	1·45	0·40	24·4	1193	None	9	—	—	—	—	—	9
14. Sengwa Wildlife Research Area, Zimbabwe	1	2·30	0·35	18·9	827	None	6	6	—	—	—	—	—

The elephant density or densities at the time of the study or studies, primary productivity (EVI), percentage tree cover, MAP (mm per year), and management interventions (none, providing water, fences, or both) for each site. The number of effects (*k*) includes the total for structure, abundance, population dynamics, diversity indices, and the ecological processes of trees, herbs, vertebrates and invertebrates, and soil properties for each study site.

### Analysis

For the purpose of this meta-analysis we assigned any measurement made in the presence of elephant as treatment values, and those made in elephant absence as the control values [3]. We used the Cohen’s *d* statistic to standardize the effect sizes among each of the comparisons [27]. Cohen’s *d* uses a correction factor that penalizes studies with small sample sizes and gives more weight to studies with relatively larger sample sizes [27]. We then calculated a 95% CI for separately for structure, abundance, population dynamics, diversity and ecological processes associated with trees, herbs, vertebrates, invertebrates, and for soil properties. We interpreted impact as neutral if the 95% CI overlapped with zero. If the effect size was below or above zero, and the 95% CI did not overlap with zero, we regarded the impact as either negative or positive. These calculations were performed using the *meta* package [28] in R [29] and we assigned study identity as random effect to account for multiple responses from the same study [30].

We plotted the frequency distributions of the individual effects (*k*) separately for trees, herbs, vertebrates, invertebrates and soil properties and set the bin size width of each distribution following Sturges’ rule. We used Generalized Linear Mixed-effects models (GLMMs) to evaluate if direct and indirect elephant effects can be explained by elephant densities, the time (in years) that elephants were excluded, primary productivity, mean annual precipitation, tree canopy cover, and management interventions (i.e. presence of artificial water and fences) using the *lme4* package [31]. All these selected explanatory variables (S1 Table) were included in different combinations in the GLMMs [32] and we assigned study identity as a random effect to account for dependencies among multiple responses from the same study [33]. A multi-model selection procedure selected the best models by ranking the candidate GLMMs by their AIC and Akaike weights (ω_i_) [32] and the strength of support for the best and alternate models with AIC differences (ΔAIC) in the *MuMIn* package [34] (R-scripts are provided in S2 Appendix).

## Results

The initial dataset consisted of 367 peer-reviewed papers published in 88 journals (see S3 Appendix for complete reference list). The first paper appeared before 1950s from the Budongo rain forest in Uganda. Over the following 68 years, some 80 sites across the sub-Saharan savannas were subjected to a study on elephant impact. The most studies came from the Kruger National Park in South Africa that had 56 papers published on elephant impact since 1969. Thirty-three sites across Africa had only a single paper published on impact. The number of papers on elephant impact steadily increased each year since the first one in 1947, and the papers used in our meta-analysis only started appearing after 1984 (S1 Fig).

**Fig 1:**
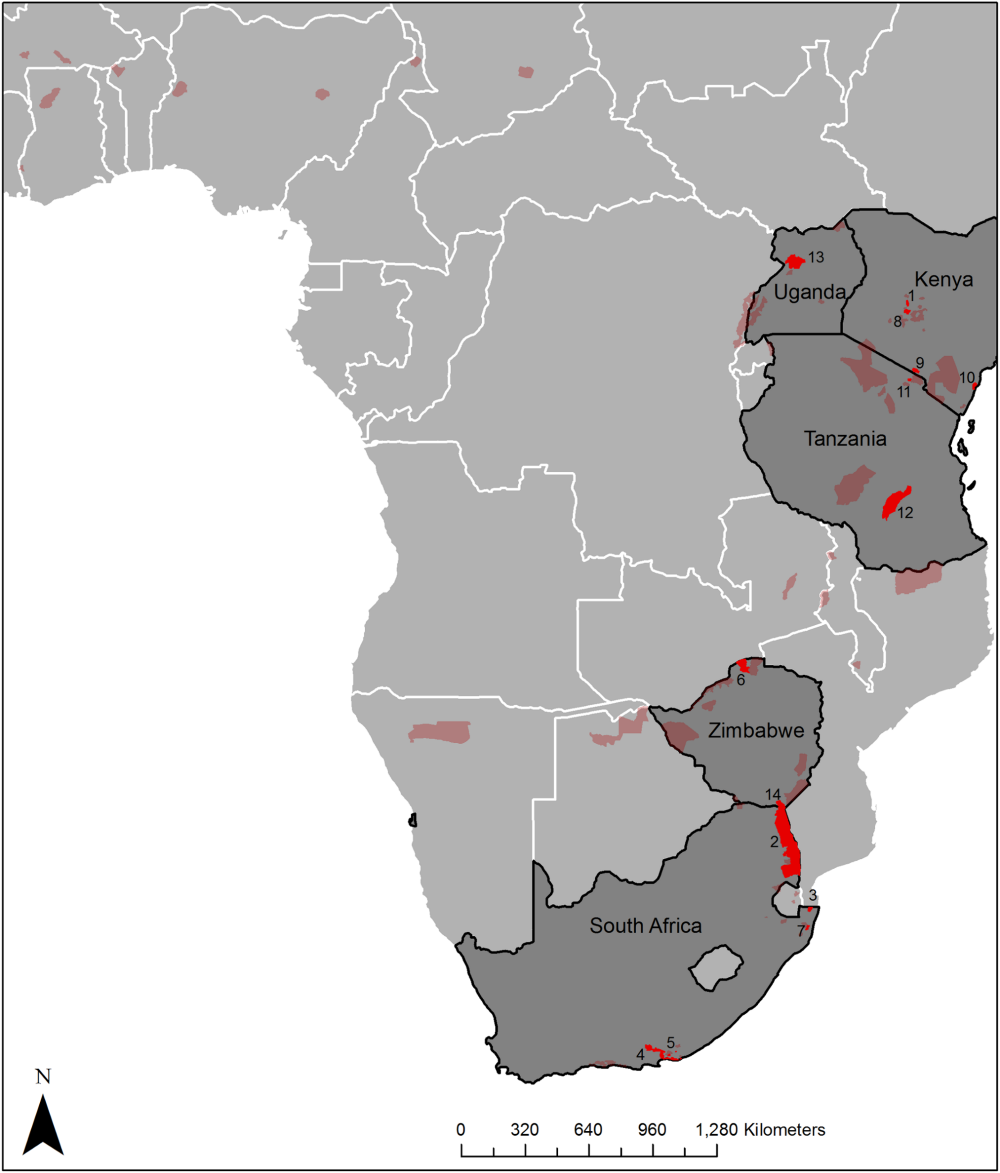
Map of sub-Saharan Africa showing the distribution of the 80 sites with elephant populations where studies were done to determine the effect elephant had for other species. The study areas in bright red and with numbers assigned to them were included in the meta-analysis. Those sites in a lighter red had papers published on elephant impact, but the papers did not meet the criteria that we have set for inclusion in the meta-analysis. The shapefiles for the protected areas were sourced from the World Database on Protected Areas (https://www.protectedplanet.net/).

Our final selection of papers came from fourteen study sites distributed across five countries, with five sites in South Africa, four in Kenya, two in Zimbabwe and Tanzania, and one in Uganda (Fig 1).

**Fig 2:**
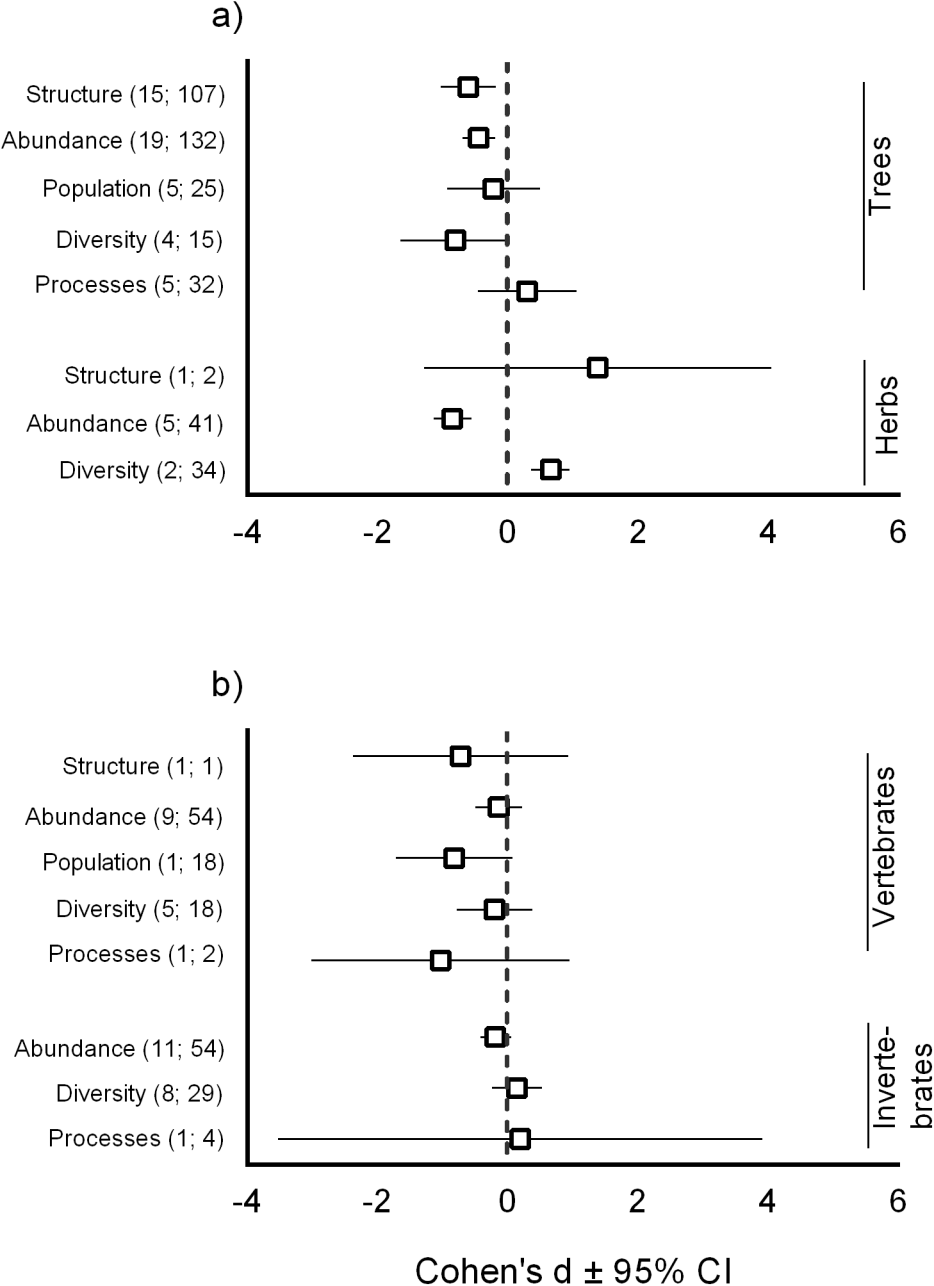
**The elephant effect size using the Cohen’s d ± 95% CI statistic separately for the structure, abundance, population dynamics, diversity and the associated ecological processes of trees and herbs (a) and vertebrates and invertebrates (b).** The first value in the bracket indicates the number of papers (*n*) from which we extracted the variables, and the second is the number of variables (*k*) used in calculating the effect sizes.

In total, we extracted 636 individual comparisons (*k*) between elephant presence and absence from the 51 papers (*n*) (Table 1). Elephants had a negative impact on tree structure and abundance, and a neutral effect on tree diversity, tree population and the associated ecological processes (Fig 2A). Herb structure was not affected by elephants, whereas diversity was positively and their abundance negatively affected. Vertebrate structure, abundance, population level responses, diversity and the associated ecological processes did not respond to the presence of elephants (Fig 2B). Impacts were also neutral on invertebrate abundance, diversity and on the associated ecological processes (Fig 2B). Soil properties responded negatively to elephant presence.

**Fig 3:**
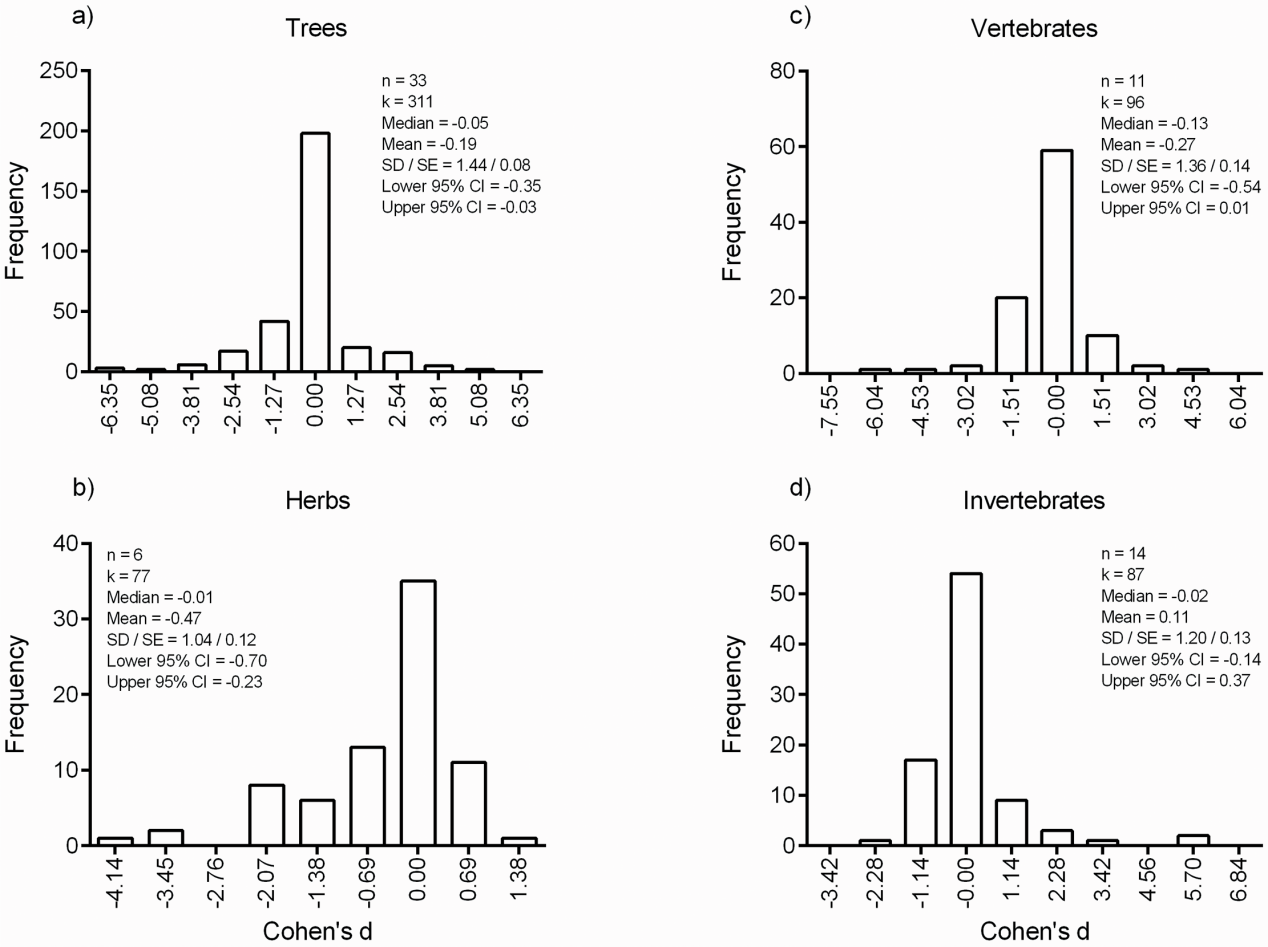
Frequency distributions of the individual elephant effects (*k*) on trees (a), herbs (b), vertebrates (c), and invertebrates (d).

Most (61·5%) of the individual measured effects (*k*) were negative, 37·4% were positive, and 1·1% had zero values. The frequency distributions of the effect sizes were unimodal. Modes centred on the zero bin classes for trees (bin width is 1·27 and effect sizes ranged from -6·18 to 5·56), herbs (bin width 0·68; ranged from -3.86 to 1·11), vertebrates (bin width 1·50; ranged from -6·43 to 4·96), invertebrates (bin width 1·14; ranged from -2·59 to 5·87), and soil properties (bin width 0·80; ranged from -4·21 to 1·13) (Fig 3A–3D and S2 Fig). The distribution of effect sizes for trees and vertebrates were almost symmetrical, left-skewed for herbs and soil properties, and right-skewed for invertebrates.

Primary productivity was the only explanatory variable included to describe the direct effects that elephants had (S2 Table). In contrast, for indirect effects, primary productivity, management interventions, elephant densities, and tree cover were all included as explanatory variables in the best set of models (S3 Table). These variables, however, explained relatively low amounts of variation in both the direct and indirect effects (marginal R^2^ for direct = 0·014 and indirect effects = 0·001 respectively).

## Discussion

Less than fifteen percent of published studies on elephant impact provided us with enough information to be included in our meta-analysis. Studies that did provide enough information illustrated that elephants have an impact on the structure and abundance of plants, with no cascading impact on small mammals, birds, herpetofauna, and invertebrate species. More importantly, we could only explain a relatively small amount of variation in elephant impact using primary productivity. Elephant numbers, study duration, rainfall, tree cover, water provision, and fences could not explain impact. In another recent review, even when elephants and other megaherbivores are absent vegetation structure changes, i.e. woody biomass and abundance increase [23], but we still lack information on the indirect effects on other species. The lack of evidence highlighted by this meta-analysis may challenge current management perspectives on how to deal with elephant impact.

We had to exclude most publications because they lacked data, had study designs without controls, or did not present proper statistical information [35]. This ultimately limited the number of samples from which we could calculate the direct and indirect effects that elephant had for other species in the African savannas. The lack of data resulted in producing relatively wide confidence intervals that overlapped with zero for some of the effect sizes, and may imply that elephants have a neutral effect on co-occurring species. These limitations in the lack of published data therefore may mask the elephant effect, and can only be resolved when we have sufficient information from which to calculate effect sizes. It calls for the participants in the peer-review process to address these limitations for future synthesis [36], to be transparent in their work [37], and to also report on the salient features of each study such as the sizes of control and treatment sites [14,20]). Replicated, randomized, and long-term ecological studies with elephant (and other mega-herbivores) exclosures in place, might in addition also provide the information from which we can evaluate elephant impact scientifically.

Savannas in Africa are heterogeneous, complex, and factors besides megaherbivores (i.e. drought, fire) can change savanna structure [38–39]. It remains contentious to separate the effects of elephants from fires, rainfall, soil mineral content, other herbivores, disease, and the impact of people [40–42]. Studies on the conversion of woodland to shrubland, or even to grassland, cannot ignore these alternative explanations as potential drivers of change in the system [8, 43–44]. To complicate matters, recent evidence indicates that elephants in arid regions suppress woody encroachment ascribed to elevated levels of atmospheric CO_2_ [15] and therefore ultimately prevent the transformation of savannas in Africa.

An added issue in interpreting elephant impact is constrained by some of the underlying assumptions. One such assumption is that statistically significant differences reflect on significant ecological differences, which may not always be the case [45–46]. Another assumption is that the measurement of impact is independent of scale. For instance, changes on an individual tree linked to elephant browsing become insignificant at the greater landscape scale, i.e. the ‘park effect’ of which elephants are part to [47–48]). A third assumption deals with a lack of ecological context, because negative values do not necessarily indicate negative impact. Lower number of species in the presence of elephants could simply be due to competitive exclusion [49], or because the community consist of pioneer and ruderal species that dominate the early stages of succession, or because of the onset of patch dynamics [50–51].

We propose that the underlying assumption for elephant impact should focus on the current philosophy of conservation, which is to restore and maintain biological diversity [52–53], and where loosing species becomes unacceptable. Our contention here is to introduce heterogeneity, as a measure of variation, to evaluate impact [54]. If elephants increase the homogeneity in the savanna landscape then impact is negative, and where heterogeneity is higher in the presence of elephants, we deduce then that they contribute positively to the maintenance of savannas in Africa. Disturbances caused by elephant foraging [11], taken as a negative impact for tree structure and numbers, establishes niches for species to occupy [14] and thereby increase species diversity and hence heterogeneity. In contrast, prevailing conditions in parts of Africa that limits elephant space use may increase homogeneity in the savanna landscape. This fits in with our lack of identifying typical explanatory covariates from which to evaluate impact. If it is not elephant numbers, savanna dynamics, management legacies, and time since afforded protection that explains elephant impact, then the management in the use of space by elephants [55] remains one of the only possibilities to negate the scale-dependent rates of disturbances of elephant [11].

This conceptual shift is relevant to conservation management that had concerns in the past on the effect that megaherbivores have on other species. These concerns motivated them to take action and to reduce numbers, i.e. through elephant culling, especially when they interpreted negative effects as negative impact [10, 56–57]). We conclude that elephants can change the plant structure and, but that this impact does not transpire into negative effects for other species that share space with them. Furthermore, in our assessment elephant density does not explain impact. We propose that the heterogeneity of the African savanna neutralizes the effect of elephants on species, and that elephants contribute positively to the maintenance of savannas [15]. Currently, there is insufficient evidence to support the notion that reducing elephant numbers *per se* will alleviate any impact.

## Supporting information

S1 Appendix The PRISMA flow diagram. (DOCX)

S2 Appendix R code for the analyses. (DOCX)

S3 Appendix Complete list of scholarly papers on elephant impact. (DOCX)

S1 Fig The number of papers published each year since 1947 on the effect that elephant had on the environment. (TIF)

S2 Fig Frequency distributions of the individual elephant effects (*k*) on soil properties. (TIF)

S1 Table Type, description, and source of the variables included in the generalized linear mixed-effects models to explain elephant impact. (DOCX)

S2 Table Selection parameters of the candidate generalized linear mixed-effects models that describe the variation in direct effects. (DOCX)

S3 Table Selection parameters of the candidate generalized linear mixed-effects models that describe the variation in indirect effects. (DOCX)
