# Supplementary material for: A systematic review of elephant impact across Africa
Source: PLoS One. 2017 Jun 7;12(6):e0178935. doi: 10.1371/journal.pone.0178935 (PMC5462389; doi:10.1371/journal.pone.0178935)
Supplement: S1 Appendix — (DOCX) [file pone.0178935.s001.docx]

S1 Appendix

Records identified through database searching (n = 367)

Additional records identified through other sources (n = 0)

Records after duplicates removed (n = 367)

Records screened (n = 367)

Records excluded (n = 294)

Full-text articles assessed for eligibility (n = 73)

Full-text articles excluded with reason (n = 22)

Studies included in quantitative synthesis (meta-analysis) (n = 51)

Identification

Screening

Eligibility

Included

**PRISMA flow diagram.**
