# Supplementary material for: A systematic review of elephant impact across Africa
Source: PLoS One. 2017 Jun 7;12(6):e0178935. doi: 10.1371/journal.pone.0178935 (PMC5462389; doi:10.1371/journal.pone.0178935)
Supplement: S2 Appendix — (DOCX) [file pone.0178935.s002.docx]

S2 Appendix

R code for meta-analysis

# Packages

library(meta)

# Set the working directory

setwd("C://R//meta_analysis//")

# Import data from .csv

meta_data<-read.csv("GLM_data_for_meta-analysis_04Aug2016.csv")

# Subset data table to run a single subset of the studies to be used

sub <- meta_data[meta_data$Main_category == "AbioticCascadingEcological_process",]

# Runs a meta-analysis using the metacont function with pooled variance and Cohen's d

meta <- metacont(sub$Exp_n, # Number of observations in experimental group.

sub$Exp_mean, # Estimated mean in experimental group.

sub$Exp_SD, # Standard deviation in experimental group.

sub$Control_n, # Number of observations in control group.

sub$Control_mean, # Estimated mean in control group.

sub$Control_SD, # Standard deviation in control group.

studlab = sub$Study_name , # An optional vector with study labels.

data=sub,

comb.fixed = T , # A logical indicating whether a random effects meta-analysis

sm="SMD", # Which summary measure is to be used for pooling of studies

pooledvar=TRUE , # Pooled variance should be used for the mean difference?

method.smd="Cohen") # Which method is used to estimate the standardised

mean difference

R code for the generalised linear mixed effects models

# Packages

library(MuMIn)

library(lme4)

# Directories for input and output

setwd("C:/R/meta_analysis/glm/")

# Read in the data

dat <- read.csv(file = "GLM_data_for_meta-analysis_04Aug2016.csv", header=T)

# Subset the data to select “Direct”

subset_dat<-dat[dat$Direct_or_cascading == "Direct",]

# Attach data

attach(subset_dat)

# GLMM

glmmFULL<-glmer(Elephant_effect~ Elephant_density +

Tree_cover + MAP + Exclosure_duration + Management_intervention + EVI +

(1|Study_ID)

, family=gaussian, data=subset_dat, na.action = na.fail)

# Model Summary

summary(glmmFULL)

# Model selection

model_selection_table <- dredge(glmmFULL,rank="AIC")
