# Supplementary material for: A systematic review of elephant impact across Africa
Source: PLoS One. 2017 Jun 7;12(6):e0178935. doi: 10.1371/journal.pone.0178935 (PMC5462389; doi:10.1371/journal.pone.0178935)
Supplement: S3 Appendix — (DOCX) [file pone.0178935.s003.docx]

S3 Appendix. The list of the 367 peer-reviewed papers published from 1947 to December 2015 that we considered in this meta-analysis.

1. Afolayan, T.A. (1973) The ecological problems facing a wildlife manager in east Africa: possible solutions to similar problems in Nigeria. The Nigerian Journal of Forestry, 3, 21-25.
2. Afolayan, T.A. (1976) Habitat utilisation by elephant in the Guinea zone. Commonwealth Forestry Review, 55, 65-71.
3. AfESG, (1993) Working group discussion three - Elephant – Habitat Working Group. Pachyderm, 17, 10-18.
4. Agnew, A.D.Q. (1968) Observations on the changing vegetation of Tsavo National Park (East). East African Wildlife Journal, 6, 75-80.
5. Anderson, G.D. & Walker, B.H. (1974) Vegetation composition and elephant damage in the Sengwa Wildlife Research Area, Rhodesia. Journal of the southern African Wildlife Management Association, 4, 1-14.
6. Asner, G.P., Levick, S.R., Kennedy-Bowdoin, T., Knapp, D.E., Emerson, R., Jacobson, J., Colgan, M.S. & Martin, R.E. (2009) Large-scale impacts of herbivores on the structural diversity of African savannas. Proceedings of the National Academy of Science, 106, 4947-4952.
7. Asner, G.P. & Levick, S.R. (2012) Landscape-scale effects of herbivore on treefall in African savannas. Ecology Letters, 15, 1211-1217.
8. Augustine, D.J. & McNaughton, S.J. (1998) Ungulate effects on the functional species composition of plant communities: herbivore selectivity and plant tolerance. Journal of Wildlife Management, 62, 1165-1183.
9. Augustine, D.J. & McNaughton, S.J. (2004) Regulation of shrub dynamics by native browsing ungulates on East African rangeland. Journal of Applied Ecology, 41, 45-58.
10. Babweteera, F., Savill, P. & Brown, N. (2007) Balanites wilsoniana: regeneration with and without elephants. Biological Conservation, 134, 40-47.
11. Bainbridge, W.R. (1965) Distribution of seed in elephant dung. Puku, 3, 173-175.
12. Banks, J.E., Jackson, C., Hannon, L.M., Thomas, C.M., Baya, A. & Njoroge, L. (2010) The cascading effects of elephant presence/absence on arthropods and an African thrush in Arabuko-Sokoke Forest, Kenya. African Journal of Ecology, 48, 1030-1038.
13. Barnes, M.E. (2001a) Seed predation, germination and seedling establishment of Acacia erioloba in northern Botswana. Journal of Arid Environments, 49, 541-554.
14. Barnes, M.E. (2001b) Effects of large herbivores and fire on the regeneration of Acacia erioloba woodlands in Chobe National Park, Botswana. African Journal of Ecology, 39, 340-350.
15. Barnes, R.F.W. (1980) The decline of the baobab tree in Ruaha National Park, Tanzania. African Journal of Ecology, 18, 243-251.
16. Barnes, R.F.W. (1983a) The elephant problem in Ruaha National Park, Tanzania. Biological Conservation, 26, 127-148.
17. Barnes, R.F.W. (1983b) Effects of elephant browsing on woodlands in a Tanzanian National Park: measurements, models and management. Journal of Applied Ecology, 20, 521-540.
18. Barnes, R.F.W. (1985) Woodland changes in Ruaha National Park (Tanzania) between 1976 and 1982. African Journal of Ecology, 23, 215-222. (missing paper)
19. Barnes, R.F.W., Barnes, K.L. & Kapela, E.B. (1994) The long-term impact of elephant browsing on baobab trees at Msembe, Ruaha National Park, Tanzania. African Journal of Ecology, 32, 177-184.
20. Baxter, P.W.J. & Getz, W.M. (2005) A model-framed evaluation of elephants effects on tree and fire dynamics in African savannas. Ecological Applications, 15, 1331-1341.
21. Baxter, P.W.J. & Getz, W.M. (2008) Development and parameterization of a rain- and fire-driven model for exploring elephant effects in African savannas. Environmental Modelling and Assessment, 13, 221-242.
22. Bell, R.H.V. (1985) Elephants and Woodlands – a reply. Pachyderm, 5, 17-18.
23. Belsky, A.J. (1984) Role of small browsing mammals in preventing woodland regeneration in the Serengeti National Park, Tanzania. African Journal of Ecology, 22, 271-279.
24. Ben-Shahar, R. (1993) Patterns of elephant damage to vegetation in northern Botswana. Biological Conservation, 65, 249-256.
25. Ben-Shahar, R. (1996a) Woodland dynamics under the influence of elephants and fire in northern Botswana. Vegetatio, 123, 153-163.
26. Ben-Shahar, R. (1996b) Do elephants over-utilize mopane woodlands in northern Botswana? Journal of Tropical Ecology, 12, 505-515.
27. Ben-Shahar, R. (1997) Elephants and woodlands in northern Botswana: how many elephants should be there? Pachyderm, 23, 41-43.
28. Ben-Shahar, R. (1998a) Changes in structure of savanna woodlands in northern Botswana following the impacts of elephants and fire. Plant Ecology, 136, 189-194.
29. Ben-Shahar, R. (1998b) Elephant density and impact on Kalahari woodland habitats. Transactions of the Royal Society of South Africa, 53, 149-155.
30. Birkett, A. (2002) The impact of giraffe, rhino and elephant on the habitat of a black rhino sanctuary in Kenya. African Journal of Ecology, 40, 276-282.
31. Birkett, A. & Stevens-Wood, B. (2005) Effect of low rainfall and browsing by large herbivores on an enclosed savannah habitat in Kenya. African Journal of Ecology, 43, 123-130.
32. Bond, W.J. (2008) What limits trees in C_4_ grasslands and savannas? Annual Review of Ecology, Evolution, and Systematics, 39, 641-659.
33. Bonnington, C., Weaver, D. & Fanning, E. (2007) Some preliminary observations on the possible effect of elephant (Loxodonta africana) disturbance on butterfly assemblages of Kilombero Valley, Tanzania. African Journal of Ecology, 46, 113-116.
34. Botes, A., McGeoch, M.A. & van Rensburg, B.J. (2006) Elephant- and human-induced changes to dung beetle (Coleoptera: Scarabaeidae) assemblages in the Maputaland Centre of Endemism. Biological Conservation, 130, 573-583.
35. Botha, J., Witkowski, E.T.F. & Shackleton, C.M. (2002) A comparison of anthropogenic and elephant disturbance on Acacia xanthophloea (fever tree) populations in the Lowveld, South Africa. Koedoe, 45, 9-18.
36. Boundja, R.P. & Midgley, J.J. (2010) Patterns of elephant impact on woody plants in the Hluhluwe-Imfolozi park, KwaZulu-Natal, South Africa. African Journal of Ecology, 48, 206-214.
37. Brahmachary, R.L. (1980) On the germination of seeds in the dung balls of the African elephant in the Virunga National Park. La Terre et la Vie, 34, 139-142.
38. Brits, J., van Rooyen, M.W. & van Rooyen, N. (2002) Ecological impact of large herbivores on the woody vegetation at selected watering points on the eastern basaltic soils in the Kruger National Park. African Journal of Ecology, 40, 53-60.
39. Buechner, H.K. & Dawkins, H.C. (1961) Vegetation change induced by elephants and fire in Murchison Falls National Park, Uganda. Ecology, 42, 752-766.
40. Buss, I.O. (1977) Management of big game with particular reference to elephants. The Malayan Nature Journal, 31, 59-71.
41. Bussmann, R.W. (1996) Destruction and management of Mount Kenya's forests. Ambio, 25, 314-317.
42. Calenge, C., Maillard, D., Gaillard, J.M., Merlot, L. & Peltier, R. (2002) Elephant damage to trees of wooded savanna in Zakouma National Park, Chad. Journal of Tropical Ecology, 18, 599-614.
43. Campbell, B.M., Butler, J.R.A., Mapaure, I., Vermeulen, S.J. & Mashove, P. (1996) Elephant damage and safari hunting in Pterocarpus angolensis woodland in northwestern Matabeleland, Zimbabwe. African Journal of Ecology, 34, 380-388.
44. Castelda, S.M., Napora, E.S., Nasseri, N.A., Vyas, D.K. & Schulte, B.A. (2010) Diurnal co-occurrence of African elephants and other mammals at a Tanzanian waterhole. African Journal of Ecology, 49, 250-252.
45. Caughley, G. (1976) The elephant problem – an alternative hypothesis. East African Wildlife Journal, 14, 265-283.
46. Chafota, J. & Owen-Smith, N. (1996) Options for the management of elephants in northern Botswana. Pachyderm, 22, 67-73.
47. Chafota, J. & Owen-Smith, N. (2009) Episodic severe damage to canopy trees by elephants: interactions with fire, frost and rain. Journal of Tropical Ecology, 25, 341-345.
48. Chamaillé-Jammes, S., Fritz, H. & Madzikanda, H. (2009) Piosphere contribution to landscape heterogeneity: a case study of remote-sensed woody cover in a high elephant density landscape. Ecography, 32, 871-880.
49. Chapman, C.A. & Chapman, L.J. (1997) Forest regeneration in logged and unlogged forests of Kibale National Park, Uganda. Biotropica, 29, 396-412.
50. Chapman, C.A., Chapman, L.J. Wrangham, R., Isabirye-Basuta, G. & Ben-David, K. (1997) Spatial and temporal variability in the structure of a tropical forest. African Journal of Ecology, 35, 287-302.
51. Chapman, L.J., Chapman, C.A. & Wrangham, R.W. (1992) Balanites wilsoniana: elephant dependent dispersal? Journal of Tropical Ecology, 8, 275-283.
52. Childes, S.L. & Walker, B.H. (1987) Ecology and dynamics of the woody vegetation on the Kalahari Sands in Hwange National Park, Zimbabwe. Vegetatio, 72, 111-128.
53. Chira, R.M. & Kinyamario, J.I. (2009) Growth response of woody species to elephant foraging in Mwea National Reserve, Kenya. African Journal of Ecology, 47, 598-605.
54. Cochrane, E.P. (2003) The need to be eaten: Balanites wilsoniana with and without elephant seed-dispersal. Journal of Tropical Ecology, 19, 579-589.
55. Coe, M. & Coe, C. (1987) Large herbivores, acacia trees and bruchid beetles. South African Journal of Science, 83, 624-635.
56. Coetzee, B.J., Engelbrecht, A.H., Joubert, S.C.J. & Retief, P.F. (1979) Elephant impact on Sclerocarya caffra trees in Acacia nigrescens tropical plains thornveld of the Kruger National Park. Koedoe, 22, 39-60.
57. Cowling, R.M., Kamineth, A., Difford, M. & Campbell, E.E. (2009) Contemporary and historical impacts of megaherbivores on the population structure of tree euphorbias in South African subtropical thicket. African Journal of Ecology, 48, 135-145.
58. Croze, H. (1974) The Seronera bull problem. II. The trees. East African Wildlife Journal, 12, 29-47. (missing paper)
59. Cumming, D.H.M., Fenton, M.B., Rautenbach, I.L., Taylor, R.D., Cumming, G.S., Cumming, M.S., Dunlop, J.M., Ford, A.G., Hovorka, M.D., Johnston, D.S., Kalcounis, M., Mahlangu, Z. & Portfors, C.V.R. (1997) Elephants, woodlands and biodiversity in southern Africa. South African Journal of Science, 93, 231-236.
60. Cumming, D.H.M. & Cumming, G.S. (2003) Ungulate community structure and ecological processes: body size, hoof area and trampling in African savannas. Oecologia, 134, 560-568.
61. Damiba, T.E. & Ables, E.D. (1994) Population characteristics and impacts on woody vegetation of elephants on Nazinga Game Ranch, Burkina Faso. Pachyderm, 18, 46-53.
62. Davis, A.L.V., Scholtz, C.H. & Swemmer, A.M. (2012) Effects of land usage on dung beetle assemblage structure: Kruger National Park versus adjacent farmland in South Africa. Journal of Insect Conservation, 16, 399-411.
63. de Beer, Y., Kilian, W., Versveld, W. & van Aarde, R.J. (2006) Elephants and low rainfall alter woody vegetation in Etosha National Park, Namibia. Journal of Arid Environments, 64, 412-421.
64. de Boer, W.F., van Oort, J.W.A., Grover, M. & Peel, M.J.S. (2015) Elephant-mediated habitat modifications and changes in herbivore species assemblages in Sabi Sand, South Africa. European Journal of Wildlife Research, doi:10.1007/s10344-015-0919-3.
65. Dharani, N., Kinyamario, J.I. & Onyari, J.M. (2006) Structure and composition of Acacia xanthophloea woodland in Lake Nakuru National Park, Kenya. African Journal of Ecology, 44, 523-530.
66. Dharani, N., Kinyamario, J.I., Wagacha, P.W. & Rodrigues, A.J. (2008) Browsing impact of large herbivores on Acacia xanthophloea Benth in Lake Nakuru National Park, Kenya. African Journal of Ecology, 47, 184-191.
67. Douglas-Hamilton, I. (1973) On the ecology and behaviour of the Lake Manyara elephants. East African Wildlife Journal, 11, 401-403.
68. Druce, D.J., Shannon, G., Page, B.R., Grant, R. & Slotow, R. (2008) Ecological thresholds in the savanna landscape: developing a protocol for monitoring the change in composition and utilization of large trees. PLoS ONE 3(12): e3979. doi:10.1371/journal.pone.0003979
69. Dublin, H.T. (1991) Dynamics of the Serengeti-Mara woodlands: An Historical Perspective. Forest and Conservation History, 35, 169-178.
70. Dublin, H.T., Sinclair, A.R.E. & McGlade, J. (1990) Elephants and fire as causes of multiple stable states in the Serengeti-Mara woodlands. Journal of Animal Ecology, 59, 1147-1164.
71. Dudley, J.P. (1999) Seed dispersal of Acacia erioloba by African bush elephants in Hwange National Park, Zimbabwe. African Journal of Ecology, 37, 375-385.
72. Dudley, J.P. (2000) Seed dispersal by elephants in semiarid woodland habitats of Hwange National Park, Zimbabwe. Biotropica, 32, 556-561.
73. Duffy, K.J., Page, B.R., Swart, J.H. & Bajic, V.B. (1999) Realistic parameter assessment for a well known elephant-tree ecosystem model reveals that limits cycles are unlikely. Ecological Modelling, 121, 115-125.
74. Duffy, K.J., van Os, R., Vos, S., van Aarde, J., Ellish, G. & Stretch, A-M.B. (2002) Estimating impact of reintroduced elephant on trees in a small reserve. South African Journal of Wildlife Research, 32, 23-29.
75. Dunham, K.M. (1989) Long-term changes in Zambezi riparian woodlands, as revealed by photopanoramas. African Journal of Ecology, 27, 263-275.
76. Eckhardt, H.C., van Wilgen, B.W. & Biggs, H.C. (2000) Trends in woody vegetation cover in the Kruger National Park, South Africa, between 1940 and 1998. African Journal of Ecology, 38, 108-115.
77. Edkins, M.T., Kruger, L.M., Harris, K. & Midgley, J.J. (2007) Baobabs and elephants in Kruger National Park: nowhere to hide. African Journal of Ecology, 46, 119-125.
78. Edroma, E.L. (1989) The response of tropical vegetation to grazing and browsing in Queen Elizabeth National Park, Uganda. Symposium of the Zoology Society London, 61, 1-13.
79. Eggeling, W.J. (1947) Observations on the ecology of the Budongo Rain Forest, Uganda. The Journal of Ecology, 34, 20-87.
80. Eltringham, S.K. (1980) A quantitative assessment of range usage by large African mammals with particular reference to the effects of elephants on trees. African Journal of Ecology, 18, 53-71.
81. Engelbrecht, A.H. (1979) Olifant invloed op Acacia nigrescens-bome in ŉ gedeelte van die Punda Milia-Sandveld van die Nationale Krugerwildtuin. Koedoe, 22, 29-37.
82. Fenton, M.B., Cumming, D.H.M., Rautenbach, I.L., Cumming, G.S., Cumming, M.S., Ford, G., Taylor, R.D., Dunlop, J., Hovorka, M.D., Johnston, D.S., Portfors, C.V., Kalcounis, M.C. & Mahlanga, Z. (1998) Bats and the loss of tree canopy in African woodlands. Conservation Biology, 12, 399-407.
83. Field, C.R. (1971) Elephant ecology in the Queen Elizabeth National Park, Uganda. East African Wildlife Journal, 9, 99-123.
84. Field, C.R. & Ross, I.C. (1976) The savanna ecology of Kidepo Valley National Park. II. Feeding ecology of elephant and giraffe. East African Wildlife Journal, 14, 1-15.
85. Fisher, J.T., Erasmus, B.F.N., Witkowski, E.T.F., van Aardt, J., Asner, G.P., Wessels, K.J. & Mathieu, R. (2014) Management approaches of conservation areas: Differences in woody vegetation structure in a private and a national reserve. South African Journal of Botany, 90, 146-152.
86. Fornara, D.A. & du Toit, J.T. (2008) Responses of woody saplings exposed to chronic mammalian herbivory in an African savanna. Ecoscience, 15, 129-135.
87. Foxcroft, L.C. & Rejmánek, M. (2007) What helps Opuntia stricta invade Kruger National Park, South Africa: Baboons or elephants? Applied Vegetation Science, 10, 265-270.
88. Franz, M., Kramer-Schadt, S., Kilian, W., Wissel, C. & Groeneveld, J. (2010) Understanding the effects of rainfall on elephant-vegetation interactions around waterholes. Ecological Modelling, 221, 2909-2917.
89. Fritz, H., Duncan, P., Gordon, I.J. & Illius, A.W. (2002) Megaherbivores influence trophic guilds structure in African ungulate communities. Oecologia, 131, 620-625.
90. Fullman, T.J. & Child, B. (2013) Water distribution at local and landscape scales affects tree utilization by elephants in Chobe National Park, Botswana. African Journal of Ecology, 51, 235-243.
91. Gadd, M.E. (2002) The impact of elephants on the marula tree Sclerocarya birrea. African Journal of Ecology, 40, 328-336.
92. Gandiwa, E., Magwati, T., Zisadza, P., Chinuwo, T. & Tafangenyasha, C. (2011) The impact of African elephants on Acacia tortilis woodland in northern Gonarezhou National Park, Zimbabwe. Journal of Arid Environments 75, 809-814.
93. Gaugris, J.Y., Matthews, W.S., van Rooyen, M.W. & Bothma, J. du P. (2004) The vegetation of Tshanini Game Reserve and a comparison with equivalent units in the Tembe Elephant Park in Maputaland, South Africa. Koedoe, 47, 9-29.
94. Gaugris, J.Y. & van Rooyen, M.W. (2010) Woody vegetation structure in conserved versus communal land in a biodiversity hotspot: A case study in Maputaland, South Africa. South African Journal of Botany, 76, 289-298.
95. Gaugris, J.Y. & van Rooyen, M.W. (2010) Effects of water dependence on the utilization pattern of woody vegetation by elephants in the Tembe Elephant park, Maputaland, South Africa. African Journal of Ecology, 48, 126-134.
96. Gaugris, J.Y. & van Rooyen, M.W. (2011) The effect of herbivores and humans on the Sand Forest species of Maputaland, northern KwaZulu-Natal, South Africa. Ecological Research, 26, 365-376.
97. Glover, J. (1963) The elephant problem at Tsavo. East African Wildlife Journal, 1, 30-39.
98. Glover, P.E. (1968) The role of fire and other influences on the savannah habitat, with suggestions for further research. East African Wildlife Journal, 6, 131-137.
99. Glover, P.E. (1970) The Tsavo and the elephants. Oryx, 10, 323-324.
100. Goheen, J.R., Keesing, F., Allan, B.F., Ogada, D. & Ostfeld, R.S. (2004) Net effects of large mammals on Acacia seedling survival in an African savanna. Ecology, 85, 1555-1561.
101. Goheen, J.R., Young, T.P., Keesing, F. & Palmer, T.M. (2007) Consequences of herbivory by native ungulates for the reproduction of a savanna tree. Journal of Ecology, 95, 129-138.
102. Goheen, J.R., Palmer, T.M., Keesing, F., Riginos, C. & Young, T.P. (2010) Large herbivores facilitate savanna tree establishment via diverse and indirect pathways. Journal of Animal Ecology, 79, 372-382.
103. Goheen, J.R. & Palmer, T.M. (2010) Defensive plant-ant stabilize megaherbivore-driven landscape change in an African savanna. Current Biology, 20, 1768-1772.
104. Gonthier, D.J. (2009) Notes on seeds deposited in elephant dung at Tarangire National Park, Tanzania. African Journal of Ecology, 47, 252-256.
105. Guldemond, R. & van Aarde, R.J. (2007) The impact of elephants on plants and their community variables in South Africa’s Maputaland. African Journal of Ecology, 45, 327-335.
106. Guldemond, R. & van Aarde, R.J. (2008) A meta-analysis of the impact of African elephants on savanna vegetation. Journal of Wildlife Management, 72, 892-899.
107. Guldemond, R. & van Aarde, R.J. (2010) The influence of tree canopies and elephants on sub-canopy vegetation in a savannah. African Journal of Ecology, 48, 180-189.
108. Guy, P.R. (1981) Changes in the biomass and productivity of woodlands in the Sengwa Wildlife Research Area, Zimbabwe. Journal of Applied Ecology, 18, 507-519.
109. Guy, P.R. (1982) Baobabs and elephants. African Journal of Ecology, 20, 215-220.
110. Guy, P.R. (1989) The influence of elephants and fire on a Brachystegia-Julbernardia woodland in Zimbabwe. Journal of Tropical Ecology, 5, 215-226.
111. Haddad, C.R., Honniball, A.S., Dippenaar-Schoeman, A.S., Slotow, R. & van Rensburg, B.J. (2009) Spiders as potential indicators of elephant-induced habitat changes in endemic sand forest, Maputaland, South Africa. African Journal of Ecology, 48, 446-460.
112. Hamandawana, H. (2012) The impacts of herbivory on vegetation in Moremi Game Reserve. Regional Environmental Change, 12, 1-15.
113. Harrington, G.N. & Ross, I.C. (1974) The savanna ecology of Kidepo Valley National Park. I. The effects of burning and browsing on the vegetation. East African Wildlife Journal, 12, 93-105.
114. Hatton, J.C., Hobsley, C. & Smart, N.O.E. (1982) Elephant poaching and vegetation changes in Uganda. Oryx, 16, 404-405.
115. Hatton, J.C. & Smart, N.O.E. (1984) The effect of long-term exclusion of large herbivores on soil nutrient status in Murchison Falls National Park, Uganda. African Journal of Ecology, 22, 23-30.
116. Hayward, M.W. & Zawadska, B. (2010) Increasing elephant Loxodonta africana density is a more important driver of change in vegetation condition than rainfall. Acta Theriologica, 55, 289-299.
117. Helm, C.V., Witkowski, E.T.F., Kruger, L., Hofmeyer, M. & Owen-Smith, N. (2009) Mortality and utilizations of Sclerocarya birrea subsp. caffra between 2001 and 2008 in the Kruger National Park, South Africa. South African Journal of Botany, 75, 475-484.
118. Helm, C., Scott, S.L. & Witkowski, E.T.F. (2011) Reproductive potential and seed fate of Sclerocarya birrea subsp. caffra (marula) in the low altitude savannas of South Africa. South African Journal of Botany, 77, 650-664.
119. Helm, C., Wilson, G., Midgley, J., Kruger, L. & Witkowski, E.T.F. (2011) Investigating the vulnerability of an African savanna tree (Sclerocarya birrea subsp. caffra) to fire and herbivory. Austral Ecology, 36, 964-973.
120. Helm, C.V. & Witkowski, E.T.F. (2012) Characterising wide spatial variation in population size structure of a keystone African savanna tree. Forest Ecology and Management, 263, 175-188.
121. Helm, C.V. & Witkowski, E.T.F. (2013) Continuing decline of a keystone tree species in the Kruger National Park, South Africa. African Journal of Ecology, 51, 270-279.
122. Hemborg, Å. & Bond, W.J. (2006) Do browsing elephants damage female trees more? African Journal of Ecology, 45, 41-48.
123. Herremans, M. (1995) Effects of woodlands modification by African elephant Loxodonta africana on bird diversity in northern Botswana. Ecography, 18, 440-454.
124. Hiscocks, K. (1999) The impact of an increasing elephant population on the woody vegetation in southern Sabi Sand Wildtuin, South Africa. Koedoe, 42, 47-56.
125. Hobbs, N.T. (1996) Modification of ecosystems by ungulates. Journal of Wildlife Management, 60, 695-713.
126. Höft, R. & Höft, M. (1995) The differential effects of elephants on rain forest communities in the Shimba Hills, Kenya. Biological Conservation, 73, 67-79.
127. Holdo, R.M. (2003) Woody plant damage by African elephants in relation to leaf nutrients in western Zimbabwe. Journal of Tropical Ecology, 19, 189-196.
128. Holdo, R.M. (2006) Tree growth in an African woodland savanna affected by disturbance. Journal of Vegetation Science, 17, 369-378.
129. Holdo, R.M. (2006) Elephant herbivory, frost damage and topkill in kalahari sand woodland savanna trees. Journal of Vegetation Science, 17, 509-518.
130. Holdo, R.M. (2007) Elephants, fire, and frost can determine community structure and composition in Kalahari woodlands. Ecological Applications, 17, 558-568.
131. Holdo, R.M., Holt, R.D. & Fryxell, J.M. (2007) Grazers, browsers, and fire influence the extent and spatial pattern of tree cover in the Serengeti. Ecological Applications, 19, 95-109.
132. Hrabar, H., Hattas, D. & du Toit, J.T. (2009) Differential effects of defoliation by mopane caterpillars and pruning by African elephants on the regrowth of Colophospermum mopane foliage. Journal of Tropical Ecology, 25, 301-309.
133. Hrabar, H. & du Toit, J.T. (2014) Interactions between megaherbivores and microherbivores: elephant browsing reduces host plant quality for caterpillars. Ecosphere, 5(1), 7 http://dx.doi.org/10.1890/ES13-00173.1
134. Ihwagi, F.W., Vollrath, F., Chira, R.M., Douglas-Hamilton, I. & Kironchi, G. (2009) The impact of elephants, Loxodonta africana, on woody vegetation through selective debarking in Samburu and Buffalo Springs National Reserves, Kenya. African Journal of Ecology, 48, 87-95.
135. Ihwagi, F.W., Chira, R.M., Kironchi, G., Vollrath, F. & Douglas-Hamilton, I. (2011) Rainfall pattern and nutrient content influences on African elephants’ debarking behaviour in Samburu and Buffalo Springs National Reserves, Kenya. African Journal of Ecology, 50, 152-159.
136. Jachmann, H. (1987) Elephants and woodland II. Pachyderm, 8, 11-12.
137. Jachmann, H. & Bell, R.H.V. (1984) Why do elephants destroy woodland? Pachyderm, 3, 9-10.
138. Jachmann, H. & Bell, R.H.V. (1985) Utilization by elephants of the Brachystegia woodlands of the Kasungu National Park, Malawi. African Journal of Ecology, 23, 245-258.
139. Jachmann, H. & Croes, T. (1991a) Effects of browsing by elephants on the Combretum/ Terminalia woodland at the Nazinga Game Ranch, Burkina Faso, West Africa. Biological Conservation, 57, 13-24.
140. Jachmann, H. & Croes, T. (1991b) Effects of browsing by elephants on Combretum- Terminalia woodland. Environmental Conservation, 18, 168-171.
141. Jacobs, O.S. & Biggs, R. (2002a) The status and population structure of the marula in the Kruger National Park. South African Journal of Wildlife Research, 32, 1-12.
142. Jacobs, O.S. & Biggs, R. (2002b) The impact of the African elephant on marula trees in the Kruger National Park. South African Journal of Wildlife Research, 32, 13-22.
143. Jacobs, S.M. & Naiman, R.J. (2008) Large African herbivore decrease herbaceous plant biomass while increasing plant species richness in a semi-arid savanna toposequence. Journal of Arid Environments, 72, 891-903.
144. Jenik, J. & Hall, J.B. (1969) The dispersal of Detarium microcarpum by elephants. The Nigerian Field, 34, 39-42.
145. Johnson, C.F., Cowling, R.M. & Phillipson, P.B. (1999) The flora of the Addo Elephant National Park, South Africa: are threatened species vulnerable to elephant damage? Biodiversity and Conservation, 8, 1447-1456.
146. Jonsson, M., Bell, D., Hjältén, J., Rooke, T. & Scogings, P.F. (2010) Do mammalian herbivores influence invertebrate communities via changes in the vegetation? Results from a preliminary survey in Kruger National Park, South Africa. African Journal of Range & Forage Science, 27, 39-44.
147. Kabigumila, J. (1993) Feeding habits of elephants in Ngorongoro Crater, Tanzania. African Journal of Ecology, 31, 156-164.
148. Kalwij, J.M., de Boer, W.F., Mucina, L., Prins, H.H.T., Skarpe, C. & Winterbach, C. (2010) Tree cover and biomass increase in a southern African despite growing elephant population. Ecological Applications, 20, 222-233.
149. Kassa, B.D., Fandohan, B., Azihou, A.F., Assogbadjo, A.E., Oduor, A.M.O., Kidjo, F.C., Babatoundé, S., Liu, J. & Kakaï, R.G. (2014) Survey of Loxodonta africana (Elephantidae)-caused bark injury on Adansonia digitata (Malacaceae) within Pendjari Biosphere Reserve, Benin. African Journal of Ecology, 52, 385-394.
150. Keesing, F. (1998) Impacts of ungulates on the demography and diversity of small mammals in central Kenya. Oecologia, 116, 381-389.
151. Kerley, G.I.H., Knight, M.H. & de Kock, M. (1995) Desertification of subtropical thicket in the Eastern Cape, South Africa: are there alternatives? Environmental Monitoring and Assessment, 37, 211-230.
152. Kerley, G.I.H. & Landman, M. (2006) The impacts of elephants on biodiversity in the eastern cape subtropical thickets. South African Journal of Science, 102, 395-402.
153. Koen, J.H. (1983) Seed dispersal by the Knysna elephants. South African Forestry Journal, 124, 56-58.
154. Kohi, E.M., de Boer, W.F., Peel, M.J.S., Slotow, R., van der Waal, C., Heitkönig, I.M.A., Skidmore, A. & Prins, H.H.T. (2011) African elephants Loxodonta africana amplify browse heterogeneity in African savanna. Biotropica, 43, 711-721.
155. Kortlandt, A. (1976) Tree destruction by elephants in Tsavo National Park and the role of man in African ecosystems. Netherlands Journal of Zoology, 26, 449-451.
156. Kupika, O.L., Kativu, S., Gandiwa, E. & Gumbie, A. (2014) Impact of African elephants on baobab (Adansonia digitata L.) population structure in northern Gonarezhou National Park, Zimbabwe. Tropical Ecology, 55, 159-166.
157. Kuiper, T.R. & Parker, D.M. (2014) Elephants in Africa: Big, grey biodiversity thieves? South African Journal of Science, 110 (3&4), 1-3.
158. Lagendijk, D.D.G., Mackey, R.L., Page, B.R. & Slotow, R. (2011) The effects of herbivory by a mega- and mesoherbivore on tree recruitment in Sand Forest, South Africa. PLoS ONE 6(3): e17983.
159. Lagendijk, D.D.G., Page, B.R. & Slotow, R. (2012) Short-term effects of single species browsing release by different-sized herbivores on sand forest vegetation community, South Africa. Biotropica 44, 63-72.
160. Lagendijk, D.D.G., Thaker, M., de Boer, W.F., Page, B.R., Prins, H.H.T. & Slotow, R. (2015) Change in mesoherbivore browsing is mediated by elephant and hillslope position. PLoS ONE 10(6): e0128340.
161. Lamprey, H.F., Glover, P.E., Turner, M.I.M. & Bell, R.H.V. (1967) Invasion of the Serengeti National Park by elephants. East African Wildlife Journal, 5, 151-166.
162. Lamprey, H.F., Halevy, G.F. & Makacha, S. (1974) Interactions between Acacia, bruchid seed beetles and large herbivores. East African Wildlife Journal, 12, 81-85.
163. Landman, M., Kerley, G.I.H. & Schoeman, D.S. (2008) Relevance of elephants herbivory as a threat to Important Plants in the Addo Elephant National Park, South Africa. Journal of Zoology, 274, 51-58.
164. Landman, M., Kerley, G.I.H. & Schoeman, D.S. (2009) Evidence-based conservation management of elephants: the case of the Important Plants in Addo Elephant National Park, South Africa. Journal of Zoology, 277, 108-110.
165. Landman, M., Schoeman, D.S. & Kerley, G.I.H. (2013) Shift in Black Rhinoceros diet in the presence of elephant: evidence for competition? PLoS One, 8, e69771.
166. Landman, M., Schoeman, D.S., Hall-Martin, A.J. & Kerley, G.I.H. (2014) Long-term monitoring reveals differing impacts of elephants on elements of a canopy shrub community. Ecological Applications, 24, 2002-2012.
167. Landman, M., Gaylard, A., Mendela, T. & Kerley, G.I.H. (2014) Impact of elephant on two woody trees, Boscia oleoides and Pappea capensis, in an arid thicket-Nama Karoo mosaic, Greater Addo Elephant National Park. Koedoe, 56, Art#1231.
168. Lawes, M.J. & Chapman, C.A. (2006) Does the herb Acanthus pubescence and/or elephants suppress tree regeneration in disturbed Afrotropical forest? Forest Ecology and Management, 221, 278-284.
169. Laws, R.M. (1970) Elephants as agents of habitat and landscape change in East Africa. Oikos, 21, 1-15.
170. Laws, R.M. (1971) The Tsavo elephants. Oryx, 11, 32-34.
171. Laws, R.M., Parker, I.S.C. & Johnstone, R.C.B. (1970) Elephants and habitats in north Bunyoro, Uganda. East African Wildlife Journal, 8, 163-180.
172. Lawton, R.M. & Gough, M. (1970) Elephants or fire – which to blame? Oryx, 10, 244-248.
173. Leuthold, W. (1977) Changes in tree populations of Tsavo East National Park, Kenya. East African Wildlife Journal, 15, 61-69.
174. Leuthold, W. (1996) Recovery of woody vegetation in Tsavo National Park, Kenya, 1970-94. African Journal of Ecology, 34, 101-112.
175. Levick, S. & Rogers, K. (2008) Patch and species specific responses of savanna woody vegetation to browser exclusion. Biological Conservation, 141, 489-498.
176. Levick, S. & Asner, G.P. (2013) The rate and spatial pattern of treefall in a savanna landscape. Biological Conservation, 157, 121-127.
177. Levick, S.R., Asner, G.P., Kennedy-Bowdoin, T. & Knapp, D.E. (2009) The relative influence of fire and herbivory on savanna three-dimensional vegetation structure. Biological Conservation, 142, 1693-1700.
178. Lewis, D.M. (1986) Disturbance effects on elephant feeding: evidence for compression in Luangwa Valley, Zambia. African Journal of Ecology, 24, 227-241.
179. Lewis, D.M. (1987) Fruiting patterns, seed germination, and distribution of Sclerocarya caffra in an elephant-inhabited woodland. Biotropica, 19, 50-56.
180. Lewis, D.M. (1991) Observations of tree growth, woodland structure and elephant damage of Colophospermum mopane in Luangwa Valley, Zambia. African Journal of Ecology, 29, 207-221.
181. Lieberman, D., Lieberman, M. & Martin, C. (1987) Notes on seeds in elephant dung from Bia National Park, Ghana. Biotropica, 19, 365-369.
182. Lindsay, K. (1986) Elephants and woodland – what are the issues? Pachyderm, 7, 16-17.
183. Lindsay, K. (1993) Elephants and habitats: the need for clear objectives. Pachyderm, 16, 34-40.
184. Lindsay, K. (1994) A reply to Spinage’s letter. Pachyderm, 18, 8-9.
185. Lindsay, K. & Olivier, R. (1984) Comments on: Why do elephants destroy woodland? Pachyderm, 4, 20.
186. Lock, J.M. (1977) Preliminary results from fire and elephant exclusion plots in Kabalega National Park, Uganda. East African Wildlife Journal, 15, 229-232.
187. Lock, J.M. (1985) Recent changes in the vegetation in Queen Elizabeth National Park, Uganda. African Journal of Ecology, 23, 63-65.
188. Lock, J.M. (1993) Vegetation change in Queen Elizabeth National Park, Uganda: 1970-1988. African Journal of Ecology, 31, 106-117.
189. Lombard, A.T., Johnson, C.F., Cowling, R.M. & Pressey, R.L. (2001) Protecting plants from elephants: botanical reserve scenarios within the Addo Elephant National Park, South Africa. Biological Conservation, 102, 191-203.
190. Loth, P.E., de Boer, W.F., Heitkönig, I.M.A. & Prins, H.H.T. (2005) Germination strategy of the East African savanna tree Acacia tortilis. Journal of Tropical Ecology, 21, 509-517.
191. MacGregor, S.D. & O’Connor, T.G. (2004) Response of Acacia tortillis to utilization by elephants in a semi-arid African savanna. South African Journal of Wildlife Research, 34, 55-66.
192. Maclean, J.E., Goheen, J.R., Doak, D.F., Palmer, T.D. & Young, T.P. (2011) Cryptic herbivores mediate the strength and form of ungulate impacts on a long-lived savanna tree. Ecology, 92, 1626-1636.
193. MacPherson, D., Hardy, M.B. & Hurt, C.R. (1994) The impact of elephant on miombo woodland in the Kasungu National Park, Malawi. Bulletin of the Grassland Society of South Africa, 5, 51-52.
194. Makhabu, S.W. & Skarpe, C. (2006) Rebrowsing by elephants three years after simulated browsing on five woody plant species in northern Botswana. South African Journal of Wildlife Research, 36, 99-102.
195. Makhabu, S.W., Skarpe, C. & Hytteborn, H. (2006) Elephant impact on shoot distribution on trees and on rebrowsing by smaller browsers. Acta Oecologica, 30, 136-146.
196. Makhabu, S.W., Skarpe, C., Hytteborn, H. & Mpofu, Z.D. (2006) The plant vigour hypothesis revisited—how is browsing by ungulates and elephant related to woody species growth rate? Plant Ecology, 184, 163-172.
197. Mapaure, I. (2001) Small-scale variations in species composition of miombo woodland in Sengwa, Zimbabwe: the influence of edaphic factors, fire and elephant herbivory. Systematics and Geography of Plants, 71, 935-947.
198. Mapaure, I. & Mhlanga, L. (1998) Elephants and woodlands: The impact of elephant damage to Colophospermum mopane on Namembere Island, Lake Kariba, Zimbabwe. The Zimbabwe Science News, 32, 15-19.
199. Mapaure, I. & Mhlanga, L. (2000) Patterns of elephant damage to Colophospermum mopane on selected islands in Lake Kariba, Zimbabwe. Kirkia, 17, 189-198.
200. Mapaure, I.N. & Campbell, B.M. (2002) Changes in the miombo woodland cover in and around Sengwa Wildlife Research Area, Zimbabwe, in relation to elephants and fire. African Journal of Ecology, 40, 212-219.
201. Mapaure, I. & Moe, S.R. (2009) Changes in the structure and composition of miombo woodlands mediated by elephants (Loxodonta africana) and fire over a 26-year period in north-western Zimbabwe. African Journal of Ecology, 47, 175-183.
202. McGeoch, M.A., van Rensburg, B.J. & Botes, A. (2002) The verification and application of bioindicators: a case study of dung beetles in a savanna ecosystem. Journal of Applied Ecology, 39, 661-672.
203. McNaughton, S.J., Ruess, R.W. & Seagle, S.W. (1988) Large mammals and process dynamics in African ecosystems. BioScience, 38, 794-800.
204. McShane, T.O. (1987) Elephant-fire relationships in Combretum/ Terminalia woodland in south-west Niger. African Journal of Ecology, 25, 79-94.
205. McShane, T.O. (1989) Some preliminary results of the relationship between soils and tree response to elephant damage. Pachyderm, 11, 29-31.
206. Mentis, M.T. (1998) Elephants, woodlands and biodiversity in southern Africa – a critique. South African Journal of Science, 94, 459.
207. Midgley, J.J. (2009) Elephant impacts to important plants in Addo Elephant National Park; comment on Landman et al. (2008). Journal of Zoology, 277, 106-107.
208. Midgley, J.J. & Joubert, D. (1991) Mistletoes, their host plants and the effects of browsing by large mammals in Addo Elephant National Park. Koedoe, 34, 149-152.
209. Midgley, J.J, Balfour, D. & Kerley, G.I. (2005) Why do elephants damage savanna trees? South African Journal of Science, 101, 213-215.
210. Midgley, J.J., Lawes, M.J. & Chamaillé-Jammes, S. (2010) Savanna woody plant dynamics: the role of fire and herbivory, separately and synergistically. Australian Journal of Botany, 58, 1-11.
211. Midgley, J.J., Gallaher, K. & Kruger, L.M. (2012) The role of the elephant (Loxodonta africana) and the tree squirrel (Paraxerus cepapi) in marula (Sclerocarya birrea) seed predation, dispersal and germination. Journal of Tropical Ecology, 28, 227-231.
212. Miller, M.F. (1994) Large African herbivores, bruchid beetles and their interactions with Acacia seeds. Oecologia, 97, 265-270.
213. Miller, M.F. (1995) Acacia seed survival, seed germination and seedling growth following pod consumption by large herbivores and seed chewing by rodents. African Journal of Ecology, 33, 194-210.
214. Miller, M.F. & Coe, M. (1993) Is it advantageous for Acacia seeds to be eaten by ungulates? Oikos, 66, 364-368.
215. Moe, S.R., Rutina, L.P., Hytteborn, H. & du Toit, J.T. (2009) What controls woodland regeneration after elephants have killed the big trees? Journal of Applied Ecology, 46, 223-230.
216. Moncrieff, G.R., Kruger, L.M. & Midgley, J.J. (2008) Stem mortality of Acacia nigrescens induced by the synergistic effects of elephants and fire in Kruger National Park, South Africa. Journal of Tropical Ecology, 24, 655-662.
217. Moncrieff, G.R., Chamaillé-Jammes, S., Higgins, S.I., O’Hara, R.B. & Bond, W.J. (2011) Tree allometries reflect a lifetime of herbivory in an African savanna. Ecology, 92, 2310-2315.
218. Moolman, H.J. & Cowling, R.M. (1994) The impact of elephant and goat grazing on the endemic flora of South African Succulent Thicket. Biological Conservation, 68, 53-61.
219. Mosugelo, D.K., Moe, S.R., Ringrose, S. & Nellemann C. (2002) Vegetation changes during a 36-year period in northern Chobe National Park, Botswana. African Journal of Ecology, 40, 232-240.
220. Mtui, D. & Owen-Smith, N. (2006) Impact of elephants (Loxodonta africana) on woody plants in Malolotja Nature Reserve, Swaziland. African Journal of Ecology, 44, 407-409.
221. Munyati, C. & Sinthumule, N.I. (2013) Assessing change in woody vegetation cover in the Kruger National Park, South Africa, using spectral mixture analysis of a Landsat TM image time series. International Journal of Environmental Studies, 70, 94-110.
222. Muoria, P.K., Gordon, I. & Oguge, N.O. (2001) Elephants as seed dispersal agents in Arabuko-Sokoke Forest, Kenya. Pachyderm, 30, 75-80.
223. Musgrave, M.K. & Compton, S.G. (1997) Effects of elephant damage to vegetation on the abundance of phytophagous insects. African Journal of Ecology, 35, 370-373.
224. Mukwashi, K., Gandiwa, E. & Kativu, S. (2012) Impact of African elephants on Baikiaea plurijuga woodland around natural and artificial watering points in northern Hwange National Park, Zimbabwe. International Journal of Environmental Sciences, 2, 1355-1368.
225. Munyathi, C. & Sinthumule, N.I. (2013) Assessing change in woody vegetation cover in the Kruger National Park, South Africa, using spectral mixture analysis of a Landsat TM image time series. International Journal of Environmental Studies, 70, 94-110.
226. Muvengwi, J., Mbiba, M. & Nyenda, T. (2015) Using branch diameter to estimate fresh biomass removal by elephants: comparison of linear and quadratic models. African Journal of Ecology, 53, 126-129.
227. Mwalyosi, R.B.B. (1981) Ecological changes in Lake Manyara National Park. African Journal of Ecology, 19, 201-204.
228. Mwalyosi, R.B.B. (1987) Decline of the Acacia tortilis in Lake Manyara National Park, Tanzania. African Journal of Ecology, 25, 51-53.
229. Mwalyosi, R.B.B. (1990) The dynamic ecology of Acacia tortilis woodland in Lake Manyara National Park, Tanzania. African Journal of Ecology, 28, 189-199.
230. Myers, N. (1973) Tsavo National Park, Kenya, and its elephants: an interim appraisal. Biological Conservation, 5, 123-132.
231. Naiman, R.J. (1988) Animal influences on ecosystem dynamics. BioScience, 38, 750-752.
232. Napier Bax, P. & Sheldrick, D.L.W. (1963) Some preliminary observations on the food of elephant in the Tsavo Royal National Park (East) of Kenya. East African Wildlife Journal, 1, 40-53.
233. Nasseri, N.A., McBrayer, L.D. & Schulte, B.A. (2011) The impact of tree modification by African elephant (Loxodonta africana) on herpetofaunal species richness in northern Tanzania. African Journal of Ecology, 49, 133-140.
234. Ndoro, O., Mashapa, C., Kativu, S. & Gandiwa, E. (2015) A comparative assessment of baobab density in northern Mana Pools National Park, Zimbabwe. African Journal of Ecology, 53, 109-111.
235. Nellemann, C., Moe, S.R. & Rutina, L.P. (2002) Links between terrain characteristics and forage patterns of elephants (Loxodonta africana) in northern Botswana. Journal of Tropical Ecology, 18, 835-844.
236. Nellis, M.D. & Bussing, C.E. (1990) Spatial variation in elephant impact on the Zambezi Teak Forest in the Chobe National Park, Botswana. Geocarto International, 2, 55-57.
237. Nellis, M.D., Lulla, K., Briggs, J.M. & Bussing, C.E. (1990) Interfacing geographic information systems and Space Shuttle photography for monitoring elephant impact in Botswana. Papers and Proceedings of Applied Geography Conferences, 13, 10-15.
238. Novellie, P. (1988) The impact of large herbivores on the grassveld in the Addo Elephant National Park. South African Journal of Wildlife Research, 18, 6-10.
239. Novellie, P., Hall-Martin, A.J. & Joubert, D. (1991) The problem of maintaining large herbivores in small conservation areas: deterioration of the grassveld in the Addo Elephant National Park. Koedoe, 34, 41-50.
240. O’Connor, T.G. (2010) Transformation of riparian forests to woodland in Mapungubwe National Park, South Africa, between 1990 and 2007. Austral Ecology, 35, 778-786.
241. O’Connor, T.G., Goodman, P.S. & Clegg, B. (2007) A functional hypothesis of the threat of local extirpation of woody plant species by elephant in Africa. Biological Conservation, 136, 329-345.
242. O’Connor, T.G. & Page, B.R. (2014) Simplification of the composition, diversity and structure of woody vegetation in a semi-arid African savanna reserve following the re-introduction of elephants. Biological Conservation, 180, 122-133.
243. Odadi, W.O., Karachi, M.K., Abdulrazak, S.A. & Young, T.P. (2011) African wild ungulates compete with or facilitate cattle depending on season. Science, 333, 1753-1755.
244. Ogada, D.L., Gadd, M.E., Ostfeld, R.S., Young, T.P. & Keesing, F. (2008) Impacts of large herbivorous mammals on bird diversity and abundance in an African savanna. Oecologia, 156, 387-397.
245. O’Kane, C.A.J., Duffy, K.J., Page, B.R. & MacDonald, D.W. (2014) Model highlights likely long-term influences of mesobrowsers versus those of elephants on woodland dynamics. African Journal of Ecology, 52, 192-208.
246. Okula, J.P. & Sise, W.R. (1986) Effects of elephant browsing on Acacia seyal in Waza National Park, Cameroon. African Journal of Ecology, 24, 1-6.
247. Omeja, P.A., Jacob, A.L., Lawes, M.J., Lwanga, J.S., Rothman, J.M., Tumwesigye, C. & Chapman, C.A. (2014) Changes in elephant abundance affect forest composition or regeneration? Biotropica, 46, 704-711.
248. Onyeanusi, A.E., Ero, I.I. & Kushi, A.A.M. (1988) Utilisation of baobab trees by elephants in Yankari Game Reserve. The Nigerian Field, 53, 163-166.
249. Or, K. & Ward, D. (2003) Three-way interactions between Acacia, large mammalian herbivores and bruchid beetles – a review. African Journal of Ecology, 41, 257-265.
250. Osborn, F.V. 2002. Elephant-induced change in woody vegetation and its impact on elephant movements out of a protected area in Zimbabwe. Pachyderm, 33, 50-57.
251. Owen-Smith, N. (2002) Credible models for herbivore-vegetation systems: towards an ecology of equations. South African Journal of Science, 98, 445-449.
252. Page, B.R. (1997) Learning about the interaction between elephants and their habitats: twenty-one years of the Tuli Elephant Project. The Rhino and Elephant Journal, 11, 31-34.
253. Palmer, T.M., Stanton, M.L., Young, T.P., Goheen, J.R., Pringle, R.M. & Karban, R. (2008) Breakdown of an ant-plant mutualism follows the loss of large herbivores from an African savanna. Science, 319, 192-195.
254. Parker, A.H. & Witkowski, E.T.F. (1999) Long-term impacts of abundant perennial water provision for game on herbaceous vegetation in a semi-arid African savanna woodland. Journal of Arid Environments, 41, 309-321.
255. Parker, D.M. & Bernard, R.T.F. (2008) Lessons from aloes in the Thicket Biome: reconstructing past elephant browsing to understand the present. South African Journal of Science, 104, 163-164.
256. Parker, D.M., Bernard, R.T.F. & Adendorff, J. (2009) Do elephants influence the organisation and function of a South African grassland? The Rangeland Journal, 31, 395-403.
257. Parker, D.M. & Bernard, R.T.F. (2009) Levels of aloe mortality with and without elephants in the Thicket Biome of South Africa? African Journal of Ecology, 47, 246-251.
258. Paugy, M., Baillon, F., Chevalier, D. & Duponnois, R. (2004) Elephants as dispersal agents of mycorrhizal spores in Burkina Faso. African Journal of Ecology, 42, 225-227.
259. Paul, J.R., Randle, A.M., Chapman, C.A. & Chapman, L.J. (2004) Arrested succession in logging gaps: is tree seedling growth and survival limiting? African Journal of Ecology, 42, 245-251.
260. Pellew, R.A.P. (1983) The impacts of elephant, giraffe and fire upon the Acacia tortilis woodlands of the Serengeti. African Journal of Ecology, 21, 41-74.
261. Penzhorn, B.L., Robbertse, P.J. & Olivier, M.C. (1974) The influence of the African elephant on the vegetation of the Addo Elephant National Park. Koedoe, 17, 137-158.
262. Plumptre, A.J. (1993) The effects of trampling damage by herbivores on the vegetation of the Parc National Des Volcans, Rwanda. African Journal of Ecology, 32, 115-129.
263. Plumptre, A.J. (1996) Modelling the impact of large herbivores on the food supply of Mountain Gorillas and implications for management. Biological Conservation, 75, 147-155.
264. Pringle, R.M. (2008) Elephants as agents of habitat creation for small vertebrates at the patch scale. Ecology, 89, 26-33.
265. Pringle, R.M., Young, T.P., Rubenstein, D.I. & McCauley, D.J. (2007) Herbivore-initiated interaction cascades and their modulation by productivity in an African savanna. Proceedings of the National Academy of Science, 104, 193-197.
266. Pringle, R.M., Kimuyu, D.M., Sesenig, R.L., Palmer, T.M., Riginos, C., Veblen, K.E. & Young, T.P. (2015) Synergistic effects of fire and elephants on arboreal animals in an African savanna. Journal of Animal Ecology, 84, 1637-1645.
267. Prins, H.H.T. & van der Jeugd, H.P. (1993) Herbivore population crashes and woodland structure in East Africa. Journal of Ecology, 81, 305-314.
268. Raymer, D. (1995) Declining riverine woodlands of Acacia xanthophloea at Lewa Downs, near Isiolo. East Africa Natural History Society Bulletin, 25, 56-59.
269. Ribeiro, N.S., Shugart, H.H. & Washington-Allen, R. (2008) The effects of fire and elephants on species composition and structure of the Niassa Reserve, northern Mozambique. Forest Ecology and Management, 255, 1626-1636.
270. Richardson-Kageler, S.J. (2004) Effects of large herbivore browsing on the functional groups of woody plants in a southern African savanna. Biodiversity and Conservation, 13, 2145-2163.
271. Robinson, J.A., Lulla, K.P., Kashiwagi, M., Suzuki, M., Nellis, M.D., Bussing, C.E., Lee Long, W.J. & McKenzie, L.J. (2001) Conservation applications of astronaut photographs of earth: Tidal-flat loss (Japan), elephant effects on vegetation (Botswana), seagrass and mangrove monitoring (Australia). Conservation Biology, 15, 876-884.
272. Ross, I.C., Field, C.R. & Harrington, G.N. (1976) The savanna ecology of Kidepo Valley National Park, Uganda. East African Wildlife Journal, 14, 35-48.
273. Ruess, R.W. & Halter, F.L. (1990) The impact of large herbivores on the Seronera woodlands, Serengeti National Park, Tanzania. African Journal of Ecology, 28, 259-275.
274. Russel, E.W. (1968) The elephant problem in the Serengeti. Oryx, 9, 404-406.
275. Rutina, L.P. & Moe, S.T. (2014) Elephant (Loxodonta africana) disturbance to riparian woodland: effects on tree-species richness, diversity and functional redundancy. Ecosystems, 17, 1384-1396.
276. Sackey, I. & Hale, W.H.G. (2008) The impact of elephants on the woody vegetation of Mole National Park, Ghana. Journal of the Ghana Science Association, 10, 28-38.
277. Salako, V.K., Azihou, A.F., Assogbadjo, A.E., Houéhanou, T.D., Kassa, B.D. & Kakaï, R.L.G. (2015) Elephant-induced damage drives spatial isolation of the dioecious palm Borassus aethiopum Mart. (Arecaceae) in the Pendjari National Park, Benin. African Journal of Ecology, doi:10.1111/aje.12253.
278. Samways, M.J. & Grant, P.B.C. (2008) Elephant impact on dragonflies. Journal of Insect Conservation, 12, 493-498.
279. Sankaran, M., Ratnam, J. & Hanan, N. (2008) Woody cover in African savannas: the role of resources, fire and herbivory. Global Ecology and Biogeography, 17, 236-245.
280. Sankaran, M., Augustine, D.J. & Ratnam, J. (2013) Native ungulates of diverse body sizes collectively regulate long-term woody plant demography and structure of a semi-arid savanna. Journal of Ecology, 101, 1389-1399.
281. Schaaf, D. (1972) Elephants, fire, and the environment. Animal Kingdom, 75, 8-15.
282. Scholtz, R., Kiker, G.A., Smit, I.P.J. & Venter, F.J. (2014) Identifying drivers that influence the spatial distribution of woody vegetation in Kruger National Park, South Africa. Ecosphere, 5, 71 <http://dx.doi.org/10.1890/ES14-000>.
283. Scogings, P.F. (2014) Large herbivores and season independently affect woody stem circumference increment in a semi-arid African savanna. Plant Ecology, 215, 1433-1443.
284. Scogings, P.F., Johansson, T., Hjälten, J. & Kruger, J. (2012) Responses of woody vegetation to exclusion of large herbivores in semi-arid savannas. Austral Ecology, 57, 56-66.
285. Scogings, P.F., Taylor, R.W. & Ward, D. (2012) Inter- and intra-plant variations in nitrogen, tannins and shoot growth of Sclerocarya birrea browsed by elephants. Plant Ecology, 213, 483-491.
286. Scogings, P.F., Hjälten, J. & Skarpe, C. (2013) Does large herbivore removal affect secondary metabolites, nutrients and shoot length in woody species in semi-arid savannas? Journal of Arid Environments, 88, 4-8.
287. Scogings, P.F., Hjälten, J., Skarpe, C., Hattas, D., Zobolo, A., Dziba, L. & Rooke, T. (2014) Nutrient and secondary metabolite concentrations in a savanna are independently affected by large herbivores and shoot growth rate. Plant Ecology, 215, 73-82.
288. Shannon, G., Druce, D.J., Page, B.R., Eckhardt, H.C., Grant, R. & Slotow, R. (2008) The utilization of large savanna trees by elephant in southern Kruger National Park. Journal of Tropical Ecology, 24, 281-289.
289. Shannon, G., Matthews, W.S., Page, B.R., Parker, G.E. & Smith, R.J. (2009) The affects of artificial water availability on large herbivore ranging patterns in savanna habitats: a new approach based on modelling elephant path distributions. Diversity and Distributions, 15, 776-783.
290. Shannon, G., Thaker, M., Vanak, A.T., Page, B.R., Grant, R. & Slotow, R. (2011) Relative impact of elephant and fire on large trees in a savanna ecosystem. Ecosystem, 14, 1372-1381.
291. Sharam, G., Sinclair, A.R.E. & Turkington, R. (2006) Establishment of broad-leaved thickets in Serengeti, Tanzania: The influence of fire, browsers, grass competition, and elephants. Biotropica, 38, 599-605.
292. Shaw, M.T., Keesing, F. & Ostfeld, R.S. (2002) Herbivory on Acacia seedlings in an East African savanna. Oikos, 98, 385-392.
293. Sheil, D. & Salim, A. (2004) Forest tree persistence, elephants, and stem scars. Biotropica, 36, 505-521.
294. Simpson, C.D. (1978) Effects of elephant and other wildlife on vegetation along the Chobe River, Botswana. Occasional Papers the Museum Texas Tech University, 48, 1-15.
295. Sinclair, A.R.E., Mduma, S.A.R., Hopcraft, J.G.C., Fryxell, J.M., Hilborn, R. & Thirgood, S. (2007) Long-term ecosystem dynamics in the Serengeti: lessons for conservation. Conservation Biology, 21, 580-590.
296. Skarpe, C., Aarrestad, P. A., Andreassen, H. P., Dhillion, S. S., Dimakatso, T., Du Toit, J. T., Halley, D. J., Hytteborn, H., Makhabu, S., Mari, M., Marokane, W., Masunga, G., Modise, D., Moe, S. R., Mojaphoko, R., Mosugelo, D., Motsumi, S., Neo-Mahupeleng, G., Ramotadima, M., Rutina, L., Sechele, L., Sejoe, T. B., Stokke, S., Swenson, J. E., Taolo, C., Vandewalle, M. & Wegge, P. (2004) The return of the giants: ecological effects of an increasing elephant population. Ambio, 33, 276-282.
297. Smallie, J.J. & O’Connor, T.G. (2000) Elephant utilization of Colophospermum mopane: possible benefits of hedging. African Journal of Ecology, 38, 352-359.
298. Smart, N.O.E., Hatton, J.C. & Spence, D.H.N. (1985) The effect of long-term exclusion of large herbivores on vegetation in Murchison Falls National Park, Uganda. Biological Conservation, 33, 229-246.
299. Smith, G.F., Klopper, R.R., Figueiredo, E., van Wyk, A.E. & Crouch, N.R. (2008) Aloes in the eastern Cape of South Africa: the value of natural history observations in biological sciences. South African Journal of Science, 104, 421-422.
300. Smith, P.P. & Shah-Smith, D.A. (1999) An investigation into the relationship between physical damage and fungal infection in Colophospermum mopane. African Journal of Ecology, 37, 27-37.
301. Spanbauer, B.R. & Adler, G.H. (2015) Seed protection through dispersal by African savannah elephants (Loxodonta africana africana) in northern Tanzania. African Journal of Ecology, doi: 10.1111/aje.12239.
302. Spinage, C.A. (1990) Botswana’s problem elephants. Pachyderm, 13, 16-21.
303. Spinage, C.A. (1994) Letters to the editor. Pachyderm, 18, 8.
304. Spinage, C.A. & Guinness, F.E. (1971) Tree survival in the absence of elephants in the Akagera National Park, Rwanda. Journal of Applied Ecology, 8, 723-728.
305. Staub, C.G., Binford, M.W. & Stevens, F.R. (2013) Elephant herbivory in Majete Wildlife Reserve, Malawi. African Journal of Ecology, 51, 536-543.
306. Steyn, A. & Stalmans, M. (2001) Selective habitat utilization and impact on vegetation by African elephant within a heterogeneous landscape. Koedoe, 44, 95-103.
307. Struhsaker, T.T., Lwanga, J.S. & Kasenene, J.M. (1996) Elephants, selective logging and forest regeneration in the Kibale Forest, Uganda. Journal of Tropical Ecology, 12, 45-64.
308. Stuart-Hill, G.C. (1992) Effects of elephants and goats on the Kaffrarian succulent thicket of the eastern Cape, South Africa. Journal of Applied Ecology, 29, 699-710.
309. Styles, C.V. & Skinner, J.D. (2000) The influence of large mammalian herbivores on growth form and utilization of mopane trees, Colophospermum mopane, in Botswana’s Northern Tuli Game Reserve. African Journal of Ecology, 38, 95-101.
310. Swanepoel, C.M. 1993. Boabab damage in Mana Pools National Park, Zimbabwe. African Journal of Ecology, 31, 220-225.
311. Swanepoel, C.M. & Swanepoel, S.M. (1986) Boabab damage by elephant in the middle Zambezi Valley, Zimbabwe. African Journal of Ecology, 24, 129-132.
312. Swart, J.H. & Duffy, K.J. (1987) The stability of a predator-prey model applied to the destruction of trees by elephants. South African Journal of Science, 83, 156-158.
313. Tafangenyasha, C. (1997) Tree loss in the Gonarezhou National Park (Zimbabwe) between 1970 and 1983. Journal of Environmental Management, 49, 355-366.
314. Tafangenyasha, C. (2001) Decline of the mountain acacia, Brachystegia glaucescens in Gonarezhou National Park, southeast Zimbabwe. Journal of Environmental Management, 63, 37-50.
315. Tchamba, M.N. (1995) The impact of elephant browsing on the vegetation in Waza National Park, Cameroon. African Journal of Ecology, 33, 184-193.
316. Tchamba, M.N. & Mahamat, H. (1992) Effects of elephant browsing on the vegetation in Kalamaloue National Park, Cameroon. Mammalia, 56, 533-540.
317. Tedonkeng Pamo, E. & Tchamba, M.N. (2001) Elephants and vegetation change in the Sahelo-Soudanian region of Cameroon. Journal of Arid Environments, 48, 243-253.
318. Teren, G. & Owen-Smith, N. (2010) Elephants and riparian woodland changes in the Linyanti region, Botswana. Pachyderm, 47, 18-25.
319. Thompson, P.J. (1975) The role of elephants, fire and other agents in the decline of a Brachystegia boehmii woodland. Journal of southern African Wildlife Management Association, 5, 11-18.
320. Thrash, I., Nel, P.J., Theron, G.K. & du P. Bothma, J. (1991) The impact of the provision of water for game on the woody vegetation around a dam in the Kruger National Park. Koedoe, 34, 131-148.
321. Trollope, W.S.W., Trollope, L.A., Biggs, H.C., Pienaar, D. & Potgieter, A.L.F. (1998) Long-term changes in the woody vegetation of the Kruger National Park, with special reference to the effects of elephants and fire. Koedoe, 41, 103-112.
322. Valeix, M., Chamaillé-Jammes, S. & Fritz, H. (2007) Interference competition and temporal niche shifts: elephants and herbivore communities at waterholes. Oecologia, 153, 739-748.
323. Valeix, M., Fritz, H., Dubois, S., Kanengoni, K., Alleaume, S. & Saïd, S. (2007) Vegetation structure and ungulate abundance over a period of increasing elephant abundance in Hwange National Park, Zimbabwe. Journal of Tropical Ecology, 23, 87-93.
324. Valeix, M., Fritz, H., Chamaillé-Jammes, S., Bourgarel, M. & Murindagomo, F. (2008) Fluctuations in abundance of large herbivore populations: insights into the influence of dry season rainfall and elephant numbers from long-term data. Animal Conservation, 11, 391-400.
325. Valeix, M., Fritz, H., Canévet, C., le Bel, S. & Madzikanda, H. (2009) Do elephants prevent other African herbivores from using waterholes in the dry season? Biodiversity and Conservation, 18, 569-576.
326. Valeix, M., Fritz, H., Sabatier, R., Murindagomo, F., Cumming, D. & Duncan, P. (2010) Elephant-induced structural changes in the vegetation and habitat selection by large herbivores in an African savanna. Biological Conservation, 144, 902-912.
327. Vanak, A.T., Shannon, G., Thaker, M., Page, B., Grant, R. & Slotow, R. (2012) Biocomplexity in large tree mortality: interactions between elephant, fire and landscape in an African savanna. Ecography, 35, 315-321.
328. Van de Koppel, J. & Prins, H.H.T. (1998) The importance of herbivore interactions for the dynamics of African savanna woodlands: an hypothesis. Journal of Tropical Ecology, 14, 565-576.
329. Van de Vijver, C.A.D.M., Foley, C.A. & Olff, H. (1999) Changes in the woody component of an East African savanna during 25 years. Journal of Tropical Ecology, 15, 545-564.
330. Van Eeden, D.G., van Rensburg, B.J., de Wijn, M. & Bothma, J. du P. (2006) The value of community-based conservation in a heterogeneous landscape: an avian case study from sand forest in Maputaland, South Africa. South African Journal of Wildlife Research, 36, 153-157.
331. Van Rensburg, B.J., McGeoch, M.A., Chown, S.L. & van Jaarsveld, A.S. (1999) Conservation of heterogeneity among dung beetles in the Maputaland Centre of Endemism, South Africa. Biological Conservation, 88, 145-153.
332. Van Rensburg, B.J., Chown, S.L. van Jaarsveld, A.S. & McGeoch, M.A. (2000a) Spatial variation and biogeography of sand forest avian assemblages in South Africa. Journal of Biogeography, 27, 1385-1401.
333. Van Rensburg, B.J., McGeoch, M.A., Matthews, W., Chown, S.L. & van Jaarsveld, A.S. (2000b) Testing generalities in the shape of patch occupancy frequency distributions. Ecology, 81, 3163-3177.
334. Van Wyk, P. & Fairall, N. (1969) The influence of the African elephant on the vegetation of the Kruger National Park. Koedoe, 12, 57-89.
335. Veblen, K.E. & Young, T.P. (2010) Contrasting effects of cattle and wildlife on the vegetation development of a savanna landscape mosaic. Journal of Ecology, 98, 993-1001.
336. Venter, S.M. & Witkowski, E.T.F. (2010) Baobab (Adansonia digitata L.) density, size-class distribution and population trends between four land-use types in northern Venda, South Africa. Forest Ecology and Management, 259, 294-300.
337. Vesey-FitzGerald, D.F. (1973a) Animal impact on vegetation and plant succession in Lake Manyara National Park, Tanzania. Oikos, 24, 314-325.
338. Vesey-FitzGerald, D. (1973b) Browse production and utilization in Tarangire National Park. East African Wildlife Journal, 11, 291-305.
339. Vesey-FitzGerald, D.F. (1974) The changing state of Acacia xanthoploea groves in Arusha National Park, Tanzania. Biological Conservation, 6, 40-47.
340. Viljoen, J.J., Reynecke, H.C., Panagos, M.D., Langbauer Jr., W.R., & Ganswindt, A. (2013) Seasonal selection preferences for woody plants by breeding herds of African elephants (Loxodonta africana) in a woodland savanna. International Journal of Ecology, 769587, 10.
341. Viljoen, P.J. (1984) Ecological role of the African elephant (Loxodanta africana) in an arid environment. South African Journal of Science, 80, 185-186.
342. Viljoen, P.J. & Bothma, J. du P. (1990) The influence of desert-dwelling elephants on vegetation in the northern Namib Desert, South-west Africa/ Namibia. Journal of Arid Environments, 18, 85-96.
343. Vogel, S.M., Henley, M.D., Rode, S.C., van der Vyver, D., Meares, K.F., Simmons, G. & de Boer, W.F. (2014) Elephant (Loxodonta africana) impact on trees used by nesting vultures and raptors in South Africa. African Journal of Ecology, doi: 10.1111/aje.12140.
344. Von Gadow, K. (1973) Observations on the utilization of indigenous trees by the Knysna elephants. Forestry in South Africa, 14, 13-17.
345. Wahungu, G.M., Mureu, L.K., Kimuyu, D.M., Birkett, A., Macharia, P.G. & Burton, J. (2011) Survival, recruitment and dynamics of Acacia drepanalobium Sjøstedt seedlings at Olpejeta Conservancy, Kenya, between 1999 and 2009. African Journal of Ecology, 49, 227-233.
346. Waithaka, J. (1993) The impact of elephant density on biodiversity in different eco-climatic zones in Kenya. Pachyderm, 16, 86-87.
347. Waithaka, J. (2001) Elephants as seed dispersal agents in Aberdare and Tsavo National Parks, Kenya. Pachyderm, 30, 70-74.
348. Waldpole, M.J., Nabaala, M. & Matankory, C. (2004) Status of the Mara woodlands in Kenya. African Journal of Ecology, 42, 180-188.
349. Watson, R.M. & Bell, R.H.V. (1969) The distribution, abundance and status of elephant in the Serengeti region of northern Tanzania. Journal of Applied Ecology, 6, 115-132.
350. Wessels, K.J., Mathieu, R., Erasmus, B.F.N., Asner, G.P., Smit, I.P.J., van Aardt, J.A.N., Fisher, J., Marais, W., Kennedy-Bowdoin, T., Knapp, D.E., Emerson, R. & Jacobson, J. (2011) Impact of communal land use and conservation on woody vegetation structure in the Lowveld savannas of South Africa. Forest Ecology and Management, 261, 19-29.
351. Western, D. (1989) The ecological role of elephants in Africa. Pachyderm, 12, 42-45.
352. Western, D. (2006) A half a century of habitat change in Amboseli National Park, Kenya. African Journal of Ecology, 48, 215-223.
353. Western, D. & van Praet, C. (1973) Cyclical changes in the habitat and climate of an East African ecosystem. Nature, 241, 104-106.
354. Western, D & Gichohi, H. (1993) Segregation effects and the impoverishment of savanna parks: the case for ecosystem viability analysis. African Journal of Ecology, 31, 269-281.
355. Western, D. & Maitumo, D. (2004) Woodland loss and restoration in a savanna park: a 20-year experiment. African Journal of Ecology, 42, 111-121.
356. Weyerhaeuser, F.J. (1985) Survey of elephant damage to baobabs in Tanzania’s Lake Manyara National Park. African Journal of Ecology, 23, 235-243.
357. White, A.M. & Goodman, P.S. (2009) Differences in woody vegetation are unrelated to use by African elephants (Loxodonta africana) in Mkhuze Game Reserve, South Africa. African Journal of Ecology, 48, 215-223.
358. Whyte, I.J. (2004) Ecological basis of the new elephant management policy for Kruger National Park and expected outcomes. Pachyderm, 36, 99-108.
359. Wilson, R.T. (1988) Vital statistics of the baobab (Adansonia digitata). African Journal of Ecology, 26, 197-206.
360. Wing, L.D. & Buss, I.O. (1970) Elephants and forests. Wildlife Monographs, 19, 1-92.
361. Wiseman, R., Page, B.R. & O’Connor, T.G. (2004) Woody vegetation change in response to browsing in Ithala Game Reserve, South Africa. South African Journal of Wildlife Research, 34, 25-37.
362. Yessoufou, K., Davies, T.J., Maurin, O., Kuzmina, M., Schaefer, H., van der Bank, M. & Savolainen, V. (2013) Large herbivores favour species diversity but have mixed impacts on phylogenetic community structure in an African savanna ecosystem. Journal of Ecology, 101, 614-625.
363. Yoaciel, S.M. (1981) Changes in the populations of large herbivores and in the vegetation community in Mweya Peninsula, Rwenzori National Park, Uganda. African Journal of Ecology, 19, 303-312.
364. Young, T.P. & Lindsay, W.K. (1988) Role of even-age population structure in the disappearance of Acacia xanthophloea woodlands. African Journal of Ecology, 26, 69-72.
365. Young, T.P., Okello, B.D., Kinyua, D. & Palmer, T.M. (1998) KLEE: a long-term multi-species herbivore exclusion experiment in Laikipia, Kenya. African Journal of Range & Forage Science, 14, 94-102.
366. Young, T.P., Stanton, M.L. & Christian, C.E. (2003) Effects of natural and simulated herbivory on spine lengths of Acacia drepanolobium in Kenya. Oikos, 101, 171-179.
367. Young, T.P., Palmer, T.M. & Gadd, M.E. (2005) Competition and compensation among cattle, zebras, and elephants in a semi-arid savanna in Laikipia, Kenya. Biological Conservation, 122, 351-359.
