# Supplementary figures and images for: A systematic review of elephant impact across Africa
Source: PLoS One. 2017 Jun 7;12(6):e0178935. doi: 10.1371/journal.pone.0178935 (PMC5462389; doi:10.1371/journal.pone.0178935)

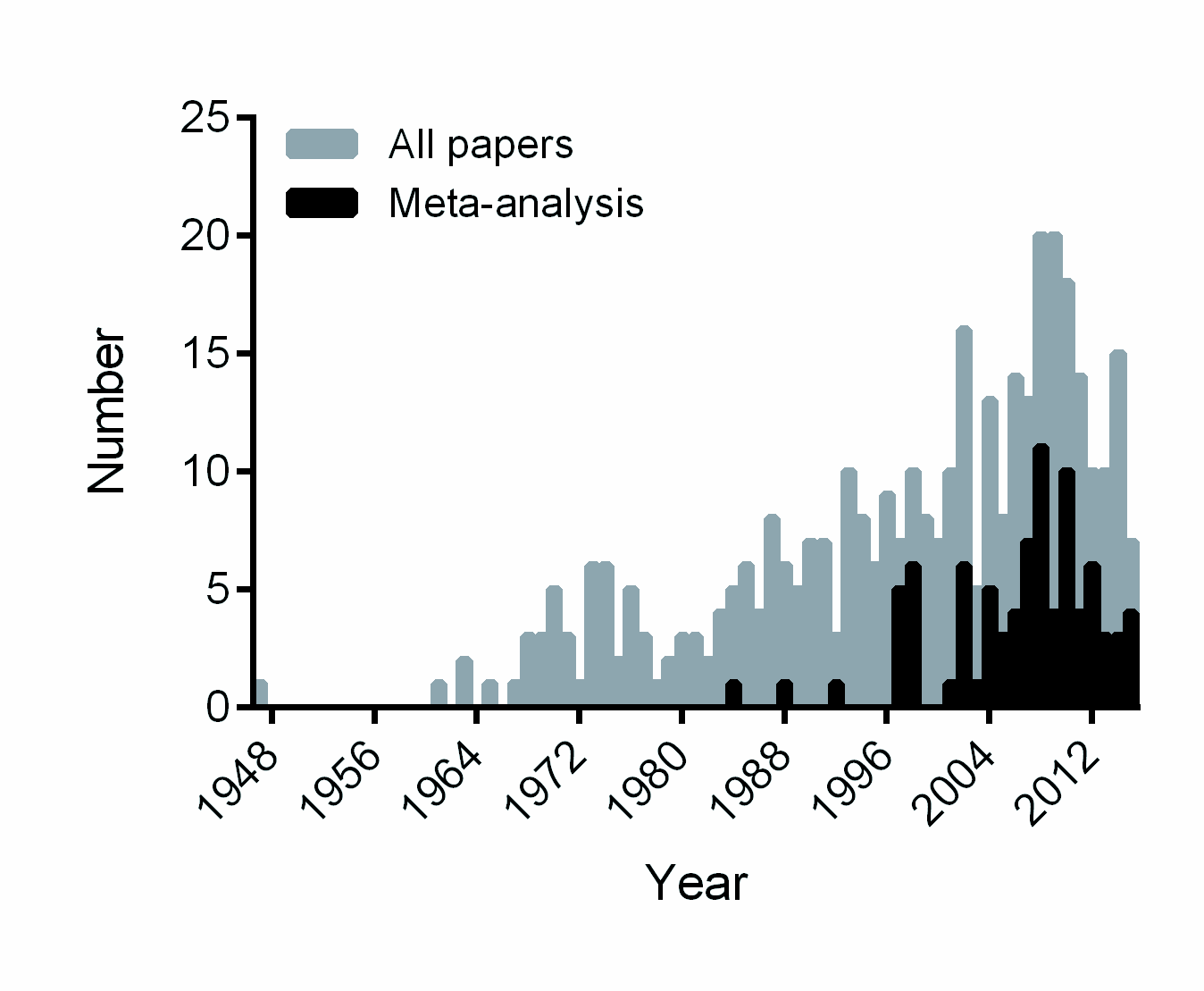

Supplement: S1 Fig — (TIF) [file pone.0178935.s004.tif]
