# Supplementary material for: A systematic review of elephant impact across Africa
Source: PLoS One. 2017 Jun 7;12(6):e0178935. doi: 10.1371/journal.pone.0178935 (PMC5462389; doi:10.1371/journal.pone.0178935)
Supplement: S1 Table — (DOCX) [file pone.0178935.s006.docx]

S1 Table. Type, description, and source of the variables included in the Generalized Linear Mixed-effects models to explain elephant effects.

| Variable | Description | Source |
| --- | --- | --- |
| Primary production | We used the Enhanced Vegetation Index (EVI) as an index of productivity, which has, along with Normalized Difference Vegetation Index (NDVI) been used widely in ecological studies and for elephants specifically [1–5]). We downloaded monthly EVI layers and calculated a long-term mean EVI (2000-2015) for each site. | <http://reverb.echo.nasa.gov/> |
| Tree cover | We downloaded annual tree cover layers (2000-2015) (MOD44B) and calculated mean tree cover for each site. | <http://reverb.echo.nasa.gov/> |
| Mean annual precipitation | We downloaded a long-term rainfall data set (WorldClim v1.4) from 1950 to 2000 [6] and calculated mean annual precipitation (MAP) for each site. | <http://www.worldclim.org/> |
| Management interventions | For each site, we did a literature search to determine if the site was fenced and if supplementary water was provided. |  |
| Elephant densities | Numbers and density of savanna elephant populations came from the African Elephant Database, our own databases [7], and publications included in this meta-analysis when the studies indicated elephant densities. | <http://www.elephantdatabase.org/> |

**References**

1. Pettorelli N, Olav Vik J, Mysterud A, Gaillard J-M, Tucker CJ, Stenseth NC (2005) Using the satellite-derived NDVI to assess ecological responses to environmental change. Trends Ecol Evol 20: 503–510.
2. Loarie SR, van Aarde RJ, Pimm SL (2009) Elephant seasonal vegetation preferences across dry and wet savannas. Biol Conserv 142: 3099–3107.
3. Roever CL, van Aarde RJ, Leggett K (2012) Functional responses in the habitat selection of a generalist mega‐herbivore, the African savannah elephant. Ecography 35: 972–982.
4. Trimble MJ, Ferreira SM, van Aarde RJ (2011) Drivers of megaherbivore demographic fluctuations: inference from elephants. J Zool 279: 18–26.
5. Wittemyer G, Rasmussen HB, Douglas-Hamilton I (2007) Breeding phenology in relation to NDVI variability in free-ranging African elephant. Ecography 30: 42–50.
6. Hijmans RJ, Cameron SE, Parra JL, Jones PG, Jarvis A (2005) Very high resolution interpolated climate surfaces for global land areas. [Int J Climatol 25: 1965–1978](http://onlinelibrary.wiley.com/doi/10.1002/joc.1276/pdf).
7. Junker J (2009) MSc thesis, University of Pretoria, Pretoria.
