# Supplementary material for: A systematic review of elephant impact across Africa
Source: PLoS One. 2017 Jun 7;12(6):e0178935. doi: 10.1371/journal.pone.0178935 (PMC5462389; doi:10.1371/journal.pone.0178935)
Supplement: S3 Table — (DOCX) [file pone.0178935.s008.docx]

S3 Table. Selection parameters of candidate generalized linear mixed-effect models that describe the variation in indirect elephant effects. The time (in years) that elephants were excluded (Duration), elephant density (Density), primary productivity (EVI), mean annual precipitation (MAP), tree canopy cover (Tree), and Management interventions (i.e. presence of artificial water and fences, Management) were included as potential explanatory variables. For each model, degrees of freedom (df), AIC (AIC), change in AIC (ΔAIC), AIC weight (AIC (wi)) are shown. We highlight the best models in bold.

| Rank | Model | df | AIC | ΔAIC | AIC _(wi)_ |
| --- | --- | --- | --- | --- | --- |
| 1. | **EVI** | **4** | **795·2** | **0·00** | **0·20** |
| 2. | **Management + Density + EVI** | **6** | **795·5** | **0·30** | **0·17** |
| 3. | **Management + Density + EVI + Tree** | **7** | **795·9** | **0·82** | **0·13** |
| 4. | **Management + EVI** | **5** | **796·5** | **1·28** | **0·11** |
| 5. | **NULL** | **3** | **796·5** | **1·37** | **0·10** |
| 6. | **Management + Density** | **5** | **796·9** | **1·79** | **0·08** |
| 7. | Density + EVI | 5 | 797·7 | 2·49 | 0·06 |
| 8. | Management | 4 | 798·1 | 2·95 | 0·05 |
| 9. | Density | 4 | 799·0 | 3·86 | 0·03 |
| 10. | EVI + Tree | 5 | 799·7 | 4·51 | 0·02 |
| 11. | Management + EVI + Tree | 6 | 799·8 | 4·62 | 0·02 |
| 12. | Density + EVI + Tree | 6 | 802·3 | 7·16 | 0·01 |
| 13. | Management + Density + EVI + Duration | 7 | 804·1 | 8·89 | 0·00 |
| 14. | EVI + Duration | 5 | 804·6 | 9·44 | 0·00 |
| 15. | Tree | 4 | 804·9 | 9·72 | 0·00 |
| 16. | Management + Density + Tree | 6 | 804·9 | 9·74 | 0·00 |
| 17. | Management + Density + EVI + Duration + Tree | 8 | 805·1 | 9·93 | 0·00 |
| 18. | Management + Density + Duration | 6 | 805·6 | 10·4 | 0·00 |
| 19. | Management + EVI + Duration | 6 | 805·8 | 10·6 | 0·00 |
| 20. | Duration | 4 | 805·9 | 10·8 | 0·00 |
| 21. | Management + Tree | 5 | 806·5 | 11·3 | 0·00 |
| 22. | Density + EVI + Duration | 6 | 806·9 | 11·7 | 0·00 |
| 23. | Density + Tree | 5 | 807·2 | 12·0 | 0·00 |
| 24. | Management + Duration | 5 | 807·4 | 12·2 | 0·00 |
| 25. | Management + EVI + Duration + Tree | 7 | 808·2 | 13·1 | 0·00 |
| 26. | Density + Duration | 5 | 808·3 | 13·1 | 0·00 |
| 27. | Management + Density + EVI + MAP | 7 | 808·4 | 13·2 | 0·00 |
| 28. | Management + Density + EVI + MAP + Tree | 8 | 808·8 | 13·6 | 0·00 |
| 29. | EVI + MAP | 5 | 808·9 | 13·7 | 0·00 |
| 30. | EVI + Duration + Tree | 6 | 808·9 | 13·7 | 0·00 |
| 31. | Management + Density + MAP | 6 | 810·1 | 14·9 | 0·00 |
| 32. | Management + EVI + MAP | 6 | 810·2 | 15·0 | 0·00 |
| 33. | MAP | 4 | 810·7 | 15·5 | 0·00 |
| 34. | Density + EVI + MAP | 6 | 811·4 | 16·2 | 0·00 |
| 35. | Density + EVI + Duration + Tree | 7 | 811·4 | 16·2 | 0·00 |
| 36. | Management + MAP | 5 | 812·1 | 16·9 | 0·00 |
| 37. | Density + MAP | 5 | 813·2 | 18·0 | 0·00 |
| 38. | EVI + MAP + Tree | 6 | 813·3 | 18·1 | 0·00 |
| 39. | Management + EVI + MAP + Tree | 7 | 813·5 | 18·3 | 0·00 |
| 40. | Management + Density + Duration + Tree | 7 | 813·5 | 18·3 | 0·00 |
| 41. | Duration + Tree | 5 | 814·3 | 19·1 | 0·00 |
| 42. | Management + Duration + Tree | 6 | 815·8 | 20·6 | 0·00 |
| 43. | Density + EVI + MAP + Tree | 7 | 815·9 | 20·8 | 0·00 |
| 44. | Management + Density + MAP + Tree | 7 | 816·0 | 20·8 | 0·00 |
| 45. | Density + Duration + Tree | 6 | 816·4 | 21·3 | 0·00 |
| 46. | Management + Density + EVI + Duration + MAP | 8 | 817·1 | 21·9 | 0·00 |
| 47. | Management + Density + EVI + Duration + MAP + Tree | 9 | 817·9 | 22·7 | 0·00 |
| 48. | EVI + Duration + MAP | 6 | 818·1 | 22·9 | 0·00 |
| 49. | MAP + Tree | 5 | 818·7 | 23·6 | 0·00 |
| 50. | Management + Density + Duration + MAP | 7 | 818·9 | 23·8 | 0·00 |
| 51. | Management + EVI + Duration + MAP | 7 | 819·2 | 24·1 | 0·00 |
| 52. | Duration + MAP | 5 | 819·9 | 24·8 | 0·00 |
| 53. | Management + MAP + Tree | 6 | 820·2 | 25·0 | 0·00 |
| 54. | Density + EVI + Duration + MAP | 7 | 820·3 | 25·2 | 0·00 |
| 55. | Density + MAP + Tree | 6 | 821·0 | 25·9 | 0·00 |
| 56. | Management + Duration + MAP | 6 | 821·1 | 25·9 | 0·00 |
| 57. | Management + EVI + Duration + MAP + Tree | 8 | 821·8 | 26·7 | 0·00 |
| 58. | Density + Duration + MAP | 6 | 822·2 | 27·1 | 0·00 |
| 59. | EVI + Duration + MAP + Tree | 7 | 822·5 | 27·3 | 0·00 |
| 60. | Density + EVI + Duration + MAP + Tree | 8 | 824·9 | 29·8 | 0·00 |
| 61. | Management + Density Duration + MAP + Tree | 8 | 825·1 | 29·9 | 0·00 |
| 62. | Duration + MAP + Tree | 6 | 827·8 | 32·6 | 0·00 |
| 63. | Management + Duration + MAP + Tree | 7 | 828·7 | 33·5 | 0·00 |
| 64. | Density + Duration + MAP + Tree | 7 | 830·0 | 34·8 | 0·00 |
